# Integrated or Independent Actions of Metformin in Target Tissues Underlying Its Current Use and New Possible Applications in the Endocrine and Metabolic Disorder Area

**DOI:** 10.3390/ijms222313068

**Published:** 2021-12-02

**Authors:** Giovanni Tulipano

**Affiliations:** Unit of Pharmacology, Department of Molecular and Translational Medicine, University of Brescia, 25123 Brescia, Italy; giovanni.tulipano@unibs.it; Tel.: +39-030-371-7510

**Keywords:** metformin, endocrinology, metabolic diseases, pituitary tumors, neuroendocrine tumors, cell metabolism, cell signaling, pyruvate, pyruvate dehydrogenase complex

## Abstract

Metformin is considered the first-choice drug for type 2 diabetes treatment. Actually, pleiotropic effects of metformin have been recognized, and there is evidence that this drug may have a favorable impact on health beyond its glucose-lowering activity. In summary, despite its long history, metformin is still an attractive research opportunity in the field of endocrine and metabolic diseases, age-related diseases, and cancer. To this end, its mode of action in distinct cell types is still in dispute. The aim of this work was to review the current knowledge and recent findings on the molecular mechanisms underlying the pharmacological effects of metformin in the field of metabolic and endocrine pathologies, including some endocrine tumors. Metformin is believed to act through multiple pathways that can be interconnected or work independently. Moreover, metformin effects on target tissues may be either direct or indirect, which means secondary to the actions on other tissues and consequent alterations at systemic level. Finally, as to the direct actions of metformin at cellular level, the intracellular milieu cooperates to cause differential responses to the drug between distinct cell types, despite the primary molecular targets may be the same within cells. Cellular bioenergetics can be regarded as the primary target of metformin action. Metformin can perturb the cytosolic and mitochondrial NAD/NADH ratio and the ATP/AMP ratio within cells, thus affecting enzymatic activities and metabolic and signaling pathways which depend on redox- and energy balance. In this context, the possible link between pyruvate metabolism and metformin actions is extensively discussed.

## 1. Introduction

Metformin (*N*,*N*-dimethylbiguanide) is considered the first-choice drug for type 2 diabetes treatment in conjunction with life style modifications, due to its efficacy, safety profile, low risk of hypoglycemia, and reduction of the risk for macrovascular complications [1,2]. Actually, pleiotropic effects of metformin have been recognized over the last two decades. Preclinical studies, retrospective studies, and clinical trials have provided evidence that this drug may have a favorable impact on health beyond its glucose-lowering activity [3]. Its beneficial effects include: reduction of food intake and body weight, reduction of the risk for cardiovascular and neuropsychiatric disorders and antineoplastic activity [4,5,6,7,8]. Moreover, preclinical investigations in animal models have suggested that metformin can slow aging and prolong lifespan [9,10,11]. Hence, despite its long history starting from its synthesis several decades ago, metformin is still an attractive research opportunity in the field of endocrine and metabolic diseases, age-related diseases, and cancer. Its mode of action in distinct cell types is still elusive.

The anticancer potential of metformin was first suggested by epidemiological data and retrospective analyses in diabetic patients, showing the association between the treatment with metformin and lower incidence of different types of solid tumors and improved survival in all examined cancer types [7,12].

Over the last decade, there has been increasing interest in the possible use of metformin to treat different types of tumors arising from endocrine glands [13].

Most of the endocrine tumors are benign and slow-growing tumors. Actually, the development of an endocrine tumor can cause hormone hyper-secretion, with severe consequences. Some tumors are more aggressive, with higher growth rates and clinically relevant tumor growth, infiltrating adjacent structures. Endocrine cancers showing metastatic spread are rare malignancies but their incidence has been reported to increase every year worldwide. Poorly differentiated aggressive cancers of the endocrine system are associated with high mortality rates and suboptimal response to treatment. Therapies for patients harboring endocrine tumors vary based on the origin of the tumor, its nature, and the tumor stage, and may include surgery, drug therapy, and radiotherapy [13,14,15].

Epidemiological studies and preclinical evidence have suggested the efficacy of metformin as a therapeutic agent for the treatment of two types of endocrine tumors, in detail differentiated thyroid cancer and pancreatic neuroendocrine tumors [13,16,17]. Furthermore, in vitro studies using cell lines and few studies in animal models have suggested to extend the efficacy of metformin to other endocrine tumors and contributed to characterize the differential effects of metformin in distinct cell types [18,19,20]. These laboratory-based data have not been confirmed by clinical evidence yet. In fact, a few clinical observations may put the efficacy of metformin into doubt [21,22].

Most of the suggested molecular targets of metformin within cells, are involved in basic functions shared by all eukaryotic cells. Nevertheless, metformin can raise differential responses in distinct cell types, both normal and tumor cells. As to the in vivo effects of metformin on tumor cells, it is worth remarking that they may be also indirect and consequent to the systemic effects of the drug, especially on plasma glucose homeostasis and related endocrine factors [19,23].

The aim of this work was to review the current knowledge on the molecular mechanisms underlying the pharmacological effects of metformin in the field of metabolic and endocrine pathologies, including endocrine tumors. Despite metformin being a drug with a long history of use, its mechanism of action has not been entirely clarified and may be regarded as still in dispute. Metformin is reported to exert pleiotropic effects through multiple pathways that can be interconnected or work independently. Moreover, metformin’s effects on target-tissues may be either direct or indirect, which means secondary to the actions on other tissues and consequent alterations at systemic level. Finally, as to the direct actions of metformin at cellular level, the intracellular milieu cooperates to cause differential responses to the drug between distinct cell types, both normal and tumor cells, despite the primary molecular target may be the same within cells.

Recent evidence either supporting or challenging the potential use of metformin to treat endocrine tumors is discussed. The preliminary review work throughout the events triggered by metformin at cellular level in distinct cell types helps to discuss the biological traits which may drive the tumor cell response to metformin. Finally, in this context, metformin is not only a potential therapeutic tool, but it also has a role as a valid tool in the basic research area aimed at identifying new targets for drug development.

## 2. Metformin Absorption, Distribution and Elimination

Metformin cannot enter cells by passive diffusion through cell membranes since it is highly hydrophilic and positively charged at physiological pH. Metformin depends on transporters to cross biological membranes. Hence, its accumulation and its effects within cells depend on the expression levels of transporters for cationic compounds at the cell surface. Understanding the biodistribution of metformin is crucial to discussing its direct- and indirect effects in specific tissues [24]. After oral administration, intestinal uptake of metformin depends on plasma membrane monoamine transporter (PMAT) and organic cation transporter subtype 1 (OCT1) [25,26]. OCT1 is also the major transporter by which metformin cross the hepatocyte plasma membrane. In humans, homozygous reduced function alleles of the gene encoding OCT1 are related to higher plasma levels of metformin and increased likelihood of intolerance symptoms to the drug, most likely due to its accumulation in the intestinal lumen [27].

After intravenous (iv) administration, metformin can be transported across the basolateral membrane of enterocytes and the significant uptake in the small intestine is dependent on OCT1 and OCT2. On the other hand, based on labeled metformin distribution into the liver, the gallbladder, and small intestine as detected by dynamic positron emission tomography, the biliary elimination of metformin and enterohepatic cycling are not major contributors for the intestinal uptake of the drug, after iv administration. Finally, OCT3 is involved in the concentration of metformin in the salivary glands, which may be regarded positively associated with dysgeusia, a common side-effect of this drug [8,28]. Multidrug and toxin extrusion proteins (MATE) are a second class of cation transporters involved in metformin passage through cell membranes. MATE1 has a role in metformin elimination from hepatocytes to the systemic circulation. Inhibition of MATE1 causes accumulation of metformin in the liver whereas it does not affect the drug distribution in the small intestine. As to the systemic elimination of metformin, this drug is not metabolized and is excreted unchanged with the urine by passive glomerular filtration and active renal secretion in the proximal tubule. OCT2 and OCT1 are involved in metformin basolateral uptake by kidney cells. MATE1 and MATE2-K (to a lower extent) contribute to its excretion into the urine [24,29,30].

Therapeutic doses of metformin as used to treat type 2 diabetes lead to steady-state plasma concentrations within the micromolar range (approximately from 5 to 20 µM). The maximal recommended dose of metformin is 35 mg/kg body weight. The high volume of distribution suggests that metformin is able to penetrate multiple tissue types. Actually, metformin can also accumulate in specific tissues overtime, leading to a higher concentration within cells compared to plasma levels. In detail, the OCT expression levels in the liver result in 3–5 times higher accumulation of metformin within the hepatocytes than the portal vein. The concentration of metformin in the gut has been observed to be 30–300 times higher than the plasma concentrations. Once inside the cells, metformin is believed to reach the mitochondrial matrix where it can accumulate, due to its positive charge and the polarization of the mitochondrial inner membrane [7,31,32].

Exploring the cellular uptake and biodistribution of metformin in vivo is also a strategy to assist the investigation aimed at a more in depth understanding of its mechanisms of action. The tissue distribution of metformin is consistent with the expression of the previously mentioned transporters; in humans, the liver, small intestine, and kidney show the highest uptake of the drug and may be suggested as primary target sites [29,33,34].

It is worth to remark that tissues other than liver and showing lower expression of transporters for cationic compounds, may be more responsive to phenformin, a derivative of metformin, than to metformin. Phenformin is more hydrophobic than metformin and can passively diffuse through cell membranes. Regarding some experimental therapeutic applications, i.e., the central control of food intake and body weight gain and antineoplastic therapy, phenformin is likely to be a more potent version of metformin. However, phenformin has been banned from clinical use due to increased risk of lactic acidosis [7,35].

## 3. Therapeutic Effects of Metformin

Metformin, along with phenformin and buformin belong to the biguanide family of anti-diabetic agents. These compounds are related to an active agent, a guanidine moiety, isolated from the plant *Galega officinalis* (French lilac), used for centuries in Europe as a herbal medicine. The guanidine moiety has been recognized as responsible for lowering blood glucose in mammals upon ingestion of the plant. Actually, early synthetic analogs of guanidine proved to be hepatotoxic and were rapidly discontinued. Biguanides were revealed to be less toxic. Nevertheless, phenformin and buformin were withdrawn from human therapy due to the occurrence of lactic acidosis and ensuing risk of mortality. The incidence of lactic acidosis with metformin at therapeutic doses is rare [7,8,36].

In type 2 diabetes, the metformin action consists mostly of decreasing glycemia without increasing plasma insulin concentrations. Metformin exerts its effects on blood glucose levels through systemic actions in the liver, where it decreases the endogenous glucose production, and to a lesser extent in the skeletal muscle, where it increases the basal glucose uptake [34,37,38,39]. More recently, the importance of local actions of metformin in the gut has been highlighted. These actions are associated with reduced glucose absorption across the gut wall into circulation, and increased secretion of glucagon-like peptide-1 (GLP-1) and peptide YY from enteroendocrine L cells [40,41].

The pharmacotherapy of polycystic ovary syndrome (PCOS) may include the use of metformin in some scenarios to treat co-existent prediabetes or type 2 diabetes, to manage menstrual irregularity, and to improve fertility. Insulin resistance and hyperandrogenism are believed to play a key role in the pathophysiology of PCOS [42,43,44,45,46,47]. To this end, metformin reduces insulin resistance and inhibits the ovarian androgen production [44,48,49,50]. In addition to lifestyle modifications, metformin can assist with the body weight loss, a key concern in women with PCOS. Indeed, this endocrine disorder is closely interrelated to obesity [51], and body weight management is a first-line therapy. Obesity has a bidirectional relationship with PCOS, with obesity increasing the PCOS prevalence and PCOS increasing the body weight gain.

Due to its modest effects, the use of metformin exclusively to cause weight loss, has not been approved in obese patients in the absence of metabolic complications. Actually, despite its administration remaining off label, metformin has been utilized in patients at high risk of metabolic complications, i.e., obese patients with evidence of pre-diabetes, and not responsive to lifestyle interventions or other medications. Finally, metformin can partially reverse drug-induced weight gain. To this regard, metformin can decrease BMI and insulin resistance in patients treated with atypical antipsychotics and can prevent the insulin-induced weight gain in patients with advanced type 2 diabetes, which require insulin therapy to achieve glycemic control [8].

Untreated type 2 diabetes is associated with an increased risk of cancer development. In diabetic patients, the treatment with metformin has been related to a lower cancer incidence compared to alternative glucose-lowering drug therapies. This clinical evidence encouraged clinical trials aimed at addressing the efficacy of a treatment with metformin in non-diabetic cancer patients, and preclinical studies aimed at elucidating the mechanisms underlying the metformin effects in the context of cancer prevention and treatment. Some trials produced promising results. Actually, large-scale studies are required to definitely assess the efficacy of metformin in different cancer types [7,12,13,52].

The anti-cancer activity of metformin in diabetic patients may be primarily linked to the reversal of hyperglycemia, hyperinsulinemia, and insulin-induced anabolism. Actually, metformin is able to cause a metabolic shift in non-diabetic patients, as well. The effects on systemic metabolism along with direct actions of metformin at cellular level, where it targets signaling pathways regulating cell metabolism and cell growth and differentiation, both cooperate to the potential anti-cancer activity of metformin [7,12].

Laboratory-based studies suggest that metformin can exert a protective effect in a variety of neurological diseases of the central nervous system, which include Parkinson’s disease, Huntington’s disease, and amyotrophic lateral sclerosis, and peripheral nervous systems, which include neuropathic pain and diabetic peripheral neuropathy [53,54]. Regarding clinical evidence, in a cohort of patients with type 2 diabetes and Huntington’s disease, the administration of metformin has been associated with an improvement of cognitive functions [55]. Many neurodegenerative diseases have been associated to metabolic failure and toxic protein aggregation in neurons. Hence, the rationale for evaluating the use of metformin to counteract neurodegeneration is related to its effects on cellular bioenergetics, mitochondrial activity, protein synthesis dysregulation, and autophagy [53,54].

Regarding psychiatric diseases, metformin has been reported to exhibit antidepressant activity. Actually, this action might be indirect and mediated by the effects on systemic metabolism. In this regard, metformin was shown to attenuate both metabolic alterations and depression-like behaviors induced by corticosterone administration in rats [56]. More in detail, metformin partially reversed the effects of corticosterone on the expression of genes related to glucose metabolism and cell sensitivity to insulin in skeletal muscle and liver, and the alterations in serum glucose and triglyceride levels, intrahepatic lipid accumulation and metabolites derived from glycolysis, TCA cycle, and gluconeogenesis.

Finally, metformin use was associated with lower cardiovascular death and morbidity compared with alternative hypoglycemic agents in the United Kingdom Prospective Diabetes Study in 1998 [57]. Actually, more recent investigations have suggested to reconsider the cardiovascular benefit of metformin monotherapy over other interventions in diabetic patients. New agents have been approved for type 2 diabetes treatment in the last twenty years and some of them have shown promising results in terms of cardiovascular benefit compared to placebo. In this regard, much research and also some changes in guidelines have involved sodium-glucose transport protein 2 (SGLT2) inhibitors and GLP-1 receptor agonists [58].

As for the mechanism underlying the cardioprotective effects of metformin in diabetic patients, it has been suggested that they can be consequent to improved vascular function and lipid profiles, and to metformin-induced weight loss [34]. They have also been attributed to some immune-modulatory actions of metformin, including suppression of the NF-kB inflammatory signaling pathway and modulatory activity on the neutrophil-to-lymphocyte ratio and plasma cytokine levels, but investigation is still ongoing to establish the actual contribution of this aspect [6,58].

## 4. Molecular Mechanisms Underlying Metformin Actions

The molecular mechanisms underlying the effects of metformin in type 2 diabetes as well as in other pathophysiological conditions, remain somewhat controversial. Controversies have been partially explained considering the variability of concentrations tested in vitro to study metformin actions at cellular and subcellular levels, and doses and routes of administration tested in animals [34,59,60]. Moreover, the concentrations of metformin used in various preclinical studies are regarded as supratherapeutic and cannot be translated into humans [13,34]. Nevertheless, it is worth to remark that some reports on the biodistribution of this drug within tissues and within subcellular compartments suggest that the steady-state plasma concentration may be significantly lower than the actual concentration at the potential intracellular targets [18,29,38,41,61].

### 4.1. Direct and Indirect Molecular Targets of Metformin within Cells

It is generally accepted that cellular bioenergetics is the primary target of metformin in normal and tumor cells. Bioenergetics is tightly coupled with signaling pathways regulating cell anabolic and catabolic functions, cell growth and differentiation, cell death, and cell survival. These pathways are indirect targets of metformin [3,12].

Metformin is regarded as a bioenergetic disruptor targeting mitochondria. The canonical mechanism of action involves the accumulation of metformin within mitochondria due to its positive charge at physiological pH value, and the inhibition of complex I (NADH:ubiquinone oxidoreductase) of the electron transport chain (ETC) [60,62,63,64,65,66]. This action results in compromised proton gradient and reduced oxygen consumption rate, with the outcome of decreased NADH oxidation and mitochondrial ATP production. The consequent shift in ATP/AMP ratio leads to a compensatory increase in glycolysis and to LKB1-mediated activation of the energy sensor AMPK, which phosphorylates a number of target proteins and plays a main role in the adaptation to energy stress conditions. If the increase of glycolysis is not enough to meet the cellular ATP requirements, the AMPK activation contributes to preserve energy by inhibiting anabolic pathways and enhancing catabolic pathways [3,12,39]. There is a hierarchy of compensatory responses in cell metabolism associated with a hierarchical activation of compartmentalized pools of AMPK, which depends on severity of nutrient or energy stress [67]. The activation of lysosomal, cytoplasmatic, and mitochondrial AMPK is triggered by different thresholds of energy depletion, which are mediated by different levels of AMP and specific metabolic intermediates. In this regard, decreasing levels of fructose-1,6 diphosphate, an intermediate of the glycolytic and gluconeogenetic pathways, leads to the activation of lysosomal AMPK, which is linked to the autophagy machinery [39,67,68].

The relevance of this canonical mechanism of action to the effects of metformin in vivo, in particular to its glucose-lowering effect, has been challenged because other targets of metformin in distinct cell types have been proposed, based on in vitro studies. Moreover, varying the concentrations of metformin in vitro may change its impact on cell metabolism and cell signaling. In summary, the molecular mechanism underlying metformin actions can be regarded as incompletely described. Nevertheless, it has been clearly established that metformin can perturb the cytosolic- and mitochondrial NAD/NADH ratio and the ATP/AMP ratio within cells, thus affecting enzymatic activities and metabolic and signaling pathways which depend on redox and energy balance [12,69].

The direct and indirect molecular targets of metformin identified in distinct cell types, include AMP deaminase, NADPH oxidase, and the glycerophosphate shuttle [12].

In rat skeletal muscle cells, metformin was found to inhibit the AMP-deaminase activity, thus increasing free AMP and ADP concentrations and activating AMPK without affecting the cellular energy charge as expressed by the ATP content. In fact, metformin was able to increase the energy formation in these cells and this finding was interpreted as consistent with the activation of lipid catabolism, which in turn can be attributed to AMPK [70].

The metformin-induced activation of AMPK was also found to mediate a decrease of reactive oxygen species (ROS) production in podocytes exposed to high glucose concentrations, through a reduction of NAD(P)H oxidase activity. NAD(P)H oxidase is the main source of ROS in podocytes in diabetes, and its production contributes to the development of diabetic nephropathy [71]. Indeed, pathological changes in podocyte structure and function induced by high glucose, play a role in the development of proteinuria in diabetic nephropathy. Metformin is believed to exert beneficial effects on diabetic kidney function, and a protective, AMPK-mediated action against damage in podocytes, is likely involved [72].

Mitochondrial glycerophosphate dehydrogenase (mGPDH) is a respiratory chain dehydrogenase. Its inhibition blocks the glycerophosphate shuttle causing accumulation of cytosolic NADH with the consequent raise of lactate/pyruvate ratio, and a more oxidized state in mitochondria with a decrease of NADH/NAD ratio. mGPDH was proposed to be a target of metformin and to play a role in gluconeogenesis inhibition in hepatocytes [73,74]. The change in mGPDH activity was also related to the antiproliferative effects of metformin in thyroid cancer cell lines [18].

### 4.2. Inhibition of Hepatic Gluconeogenesis

As for the mechanisms by which metformin inhibits hepatic gluconeogenesis, they remain debated [34,39,60] (Table 1).

In hepatocytes, the metformin-induced AMPK activation may cooperate with the gluconeogenesis regulation by altering gene expression. To this regard, metformin can reduce the expression of glucose-6-phosphatase and phosphoenolpyruvate carboxykinase (PEPCK), two key-enzymes encoded by *G6pc* and *Pck1*, respectively. There is evidence for a role of AMPK activation in the downregulation of *G6pc* and *Pck1* expression in basal non-stimulated conditions [39,75,76,77], as well as in the reversal of the cAMP-induced expression of both genes downstream of glucagon signaling pathway [78,79]. Indeed, the transcriptional complex composed by cyclic AMP response element binding protein (CREB), CREB binding protein (CBP), and CREB-regulated transcription factor 2 (CRTC2, also known as TORC2) increases the expression of both genes. The formation of the complex is stimulated by glucagon and catecholamines, and by fasting conditions. Activated AMPK has been suggested to target CRTC2 and CBP, thus causing disassembly of the transcriptional complex and reducing the *Pck1* and *G6pc* expression levels [34,39,75,76,77,80]. The activation of phsphodiesterase-4 (PDE4) is a second mechanism by which AMPK could reverse the cAMP-induced expression of *Pck1* and *G6pc* [79]. Finally, the transcriptional coactivator peroxisome proliferator-activated receptor gamma coactivator 1-alpha (PGC1-α) is a target of AMPK, and cooperates to regulate the expression of *Pck1* and *G6pc* [39,53]. PGC-1α interacts with other transcription factors to modulate various signal transduction pathways associated with mitochondrial function and cell metabolism. These factors include FOXO1, NRF-1 and NRF-2, ERRα, PPARα/δ, HNF4α, and SREBP1 [53]. In conclusion, AMPK is a valid candidate mediating the effects of metformin on the transcriptional regulation of hepatic gluconeogenesis. However, it is worth remarking that metformin concentrations higher than 0.5 mM were found to reduce *G6pc* expression to the same extent in isolated hepatocytes from AMPK-deficient mice compared with hepatocytes from control animals with normal AMPK activity [81]. Moreover, the relevance of transcriptional regulation to the reduction in gluconeogenesis induced by metformin, is a subject of debate, and cannot explain the acute changes in gluconeogenesis following drug administration [34,73,81,82].

Non-transcriptional and non-AMPK-dependent mechanisms of regulation have been proposed to mediate the metformin effects on hepatic gluconeogenesis. Two suggested mechanisms involve the cytoplasmic and mitochondrial redox state and the allosteric regulation of gluconeogenic and glycolytic enzymes, respectively [34,39].

Metformin is known to reduce the cytoplasmic redox state as estimated from the increased lactate/pyruvate ratio. To this end, metformin stimulates lactate production and increases the hyperpolarized [1-13 C]lactate conversion rate in the kidney, liver, and heart [83]. On the other hand, the effects of metformin on the mitochondrial redox state, are concentration-dependent and biphasic. When millimolar metformin concentrations are tested in vitro, the inhibition of complex I of the respiratory chain causes a decrease of ATP production and a more reduced mitochondrial redox state as estimated from the increased 3-hydroxybutyrate/acetoacetate ratio [62,64,84,85]. Actually, these concentrations may be regarded as supratherapeutic. Micromolar metformin concentrations, which are closer to clinically relevant metformin concentrations, caused a more oxidized state in the mitochondria with a lower 3-hydroxybutyrate/acetoacetate ratio, associated with a negligible ATP depletion [73,74]. Two different shuttle systems cooperate to transfer reducing equivalents from cytoplasm to mitochondria, thus avoiding depletion of NAD that is consumed in the glycolytic pathway: the glycerophosphate shuttle and the malate-aspartate shuttle. The decrease in the mitochondrial NADH/NAD ratio caused by low metformin concentrations, has been associated with a direct inhibitory activity exerted by metformin on mGPDH and the consequent block of the glycerophosphate shuttle [73,74]. Actually, an indirect inhibition of the malate-aspartate shuttle as a consequence of the mitochondrial depolarization caused by metformin is also possible [39,69].

An attenuated transfer of reducing equivalents from the cytoplasm to mitochondria due to mGPDH inhibition in hepatocytes, can account for a redox-dependent inhibition of gluconeogenesis from reduced substrates (glycerol and lactate). Based on various studies, this mechanism has been suggested to mediate the metformin therapeutic effect in type 2 diabetes [34,73]. Actually, this conclusion is still in dispute and the proposed mGPDH-dependency of metformin action in the liver, has been questioned by some reports [39,69,86]. Indeed, while causing a more oxidized redox state in mitochondria and a more reduced cytoplasmic redox state, metformin was suggested to inhibit gluconeogenesis from both reduced and oxidized substrates [39,87]. Moreover, Calza and coworkers did not observe any change in hepatic glucose production from lactate in response to metformin at therapeutic concentrations, in perfursed rat liver [88]. Finally, some groups have not observed an inhibitory effect of metformin on mGPDH activity in pancreatic beta-cells and liver cells [69,86].

The relevance of mGPDH inhibition to the therapeutic effects of metformin is also challenged by the expression of this enzyme throughout the body. Based on the effects of total-body knockout of mGPDH in mice, MacDonald and coworkers suggested that adverse effects in tissues where the level of mGPD is much higher than that in the liver could prevent the therapeutic administration of metformin in type 2 diabetes, if it actually inhibited mGPDH [86]. However, according to other researchers, this conclusion does not consider the tissue distribution of metformin, which is linked to the expression of specific transporters for cationic compounds at the cell surface [34]. As reported in a previous section, metformin primarily accumulates in the liver, kidney, and small intestine.

An alternative explanation of metformin action on hepatic gluconeogenesis involves the allosteric or substrate-dependent regulation of gluconeogenic and glycolytic enzymes: according to a metabolite perspective, micromolar concentrations of metformin could inhibit hepatic gluconeogenesis in a redox-independent manner, causing challenges in the levels of metabolites which are allosteric effectors at phosphofructokinase-1 (PFK1) and/or fructose bisphosphatase-1 (FBP1), with the outcome of a preferential partitioning to glycolysis through PFK1 activation and/or FBP1 inhibition [39,69]. Allosteric inhibitors of FBP1 include AMP and fructose 2,6-P_2_. PFK1 is activated by AMP, fructose 2,6-P_2_, fructose 1,6-P_2_, glucose 1,6-P_2_, and inorganic phosphate (P_i_), and inhibited by ATP, citrate, and glycerol-3-phosphate. The mild energetic stress induced by metformin in liver is believed to cause an increase in AMP concentration that in turn will lead to the allosteric inhibition of FBP1. The subsequent accumulation of fructose 1,6-P_2_ will contribute to increase the glycolytic flux by activating PFK1 [39,89].

The activation of PFK1 induced by altered levels of its allosteric effectors, has been proposed to explain the glucose 6-P lowering effect of therapeutically relevant doses of metformin in hepatocytes exposed to high glucose, due to the enhancement of the glycolytic flux downstream of glucose phosphorylation, in an AMPK-independent manner [90]. In this regard, in contrast with the proposed inhibitory activity of metformin on mGPDH, the overexpression of mGPDH in rat- or mouse hepatocytes mimicked the glucose 6-P lowering effect and also the *G6pc* gene repression by metformin. Indeed, the increase in mGPDH activity was associated with a reduction of glycerol-3-phosphate, which is an allosteric inhibitor of PFK1 [69,90].

The action of metformin on the allosteric regulation of the glycolytic pathway also suggests a second mechanism underlying the downregulation of *G6pc* expression, in addition to the previously mentioned AMPK-dependent mechanisms. Among the transcription factors regulating the *G6pc* expression, the carbohydrate-response element binding-protein (ChREBP) is activated by the increase of some cellular phosphorylated intermediates of glucose metabolism. In this context, the ChREBP recruitment to G*6pc* gene promoter was shown to be inhibited by metformin, which lowered the cellular glucose 6-P and fructose 2,6-P_2_ levels [90,91].

Finally, AMP is not only an allosteric effector of PFK1 and FBP1. The mild elevation in AMP levels in hepatocytes caused by metformin concentrations of therapeutic relevance, results in the inhibition of adenylyl cyclase activity and, as a consequence, glucagon signaling. Such alteration can contribute to the amelioration of glycemic control by reducing the hepatic response to a counter-regulatory hormone which antagonizes the insulin action [78]. Actually, this hypothesis has been challenged by some studies showing that metformin did not significantly affect the cAMP levels [73,75,81], and a clinical trial showing that metformin did not suppress glucagon-dependent hepatic glucose production in prediabetic subjects [82].

### 4.3. Other Actions Related to Metformin Benefits for Metabolic Health

Multiple mechanisms and various target-tissues underlie the metformin benefits for metabolic health (Table 2). The exploration of these mechanisms mediated by direct and indirect effects, has gained considerable attention in recent years in the attempt to identify new possible pharmacologic strategies for diabetes, obesity, and age-related morbidities to promote healthy aging [23]. Preclinical and clinical evidence supporting the potential “anti-aging” activity of metformin even in the absence of type 2 diabetes has been comprehensively reviewed in 2020 [92].

In addition to its effects on hepatic glucose production, metformin can increase the basal glucose uptake in skeletal muscle cells, through an AMPK-dependent mechanism [37,38,93]. More recently, metformin concentrations close to therapeutic plasma concentrations were shown to suppress the expression of the branched-chain amino acid (BCAA) catabolic enzyme BC-alpha-keto acid dehydrogenase E1a (BCKDHa) in differentiated C2C12 myotubes. Supratherapeutic concentrations suppressed the expression of a second enzyme involved in BCAA catabolism, beyond upregulating PGC-1α expression and affecting the mitochondrial respiratory chain. These data suggest that low metformin concentrations may primarily alter BCAA catabolism in skeletal muscle, with a possible outcome on circulating BCAA levels and glucose utilization [94].

There is consistent evidence that, after oral administration, the accumulation of metformin within enterocytes contributes significantly to its plasma glucose-lowering activity [41,95,96]. Indeed, delayed-release formulations of metformin have been associated to a decrease in plasma glucose levels without any increase in circulating metformin levels, suggesting a local action in the gut [97]. Metformin enters the enterocytes by organic cation transporter 1 (OCT1) and causes an increase of glucose uptake [25,26,98]. Then, glucose is utilized locally in an anaerobic manner driving lactate production in enterocytes, with the outcome of a reduced glucose bioavailability [99]. Moreover, hepatocytes use lactate to make glucose at the cost of energy expenditure, creating a futile intestine–liver cycle [95].

The local action of metformin in the gut is not limited to changes in the enterocyte metabolism. Metformin can also reshape the gut microbiota through interacting with different bacteria. Both animal and human studies confirmed that metformin can alter the relative abundance of multiple bacterial strains with favorable consequences on glycemic control and appetite [8]. In this regard, short chain fatty acids (SCFAs) such as acetate, propionate, and butyrate are important products of gut microbiota, whose levels are thought to contribute to the control of glucose homeostasis, appetite via the incretin system, and body weight gain [100]. In a mouse model of type 2 diabetes and obesity and in mice made obese by a high-fat diet, metformin was found to increase the relative number of SCFA-producing bacteria in the gut microbiota and to effectively restore the intestinal SCFA content [101,102]. In the same animals, metformin reduced food intake, body weight, plasma glucose levels, and inflammation.

Finally, it is worth remarking that metformin accumulation in the intestinal lumen and the increased lactate production in the intestinal mucosa, are regarded as responsible of some gastrointestinal side-effects and intolerance symptoms associated with metformin oral administration [27,103,104]. These side-effects may contribute to the weight loss observed with metformin therapy. Actually, more complex mechanisms by which metformin may suppress appetite and reduce body weight, have been suggested. They include various actions in the central nervous system (CNS), in detail at hypothalamic level, and again in the gut [8]. Metformin was unexpectedly found to suppress the hypothalamic AMPK activity, in agreement with an anorexigenic effect [8,105,106]. As to the gut, metformin was shown to reduce bile acid absorption through a signaling pathway which involves a crosstalk between AMPK and farnesoid X receptor [107]. In turn, this action is thought to have a secondary effect on the secretion of appetite-suppressing enteric peptides, such as GLP-1 and peptide YY [8,108]. Moreover, metformin administration was associated with increased circulating levels of growth differentiation factor-15 (GDF15) and increased GDF15 expression in the intestine and kidney. GDF15 acts through a receptor complex expressed in the hindbrain, through which it suppresses food intake. Hence, it can mediate the effects of metformin on energy balance and body weight, whereas it is not required for the plasma glucose-lowering effect [34,109,110,111]. In summary, gut hormones and peripheral metabolites both contribute to the regulation of plasma glucose- and body weight homeostasis by metformin [40,96,112].

The role of GDF15 as an endocrine mediator of metformin actions in the CNS has been characterized in the last couple of years and is worth being further discussed. GDF15 is a member of the transforming growth factor-beta (TGF-β) superfamily. It is produced by various cells in the body and its circulating concentrations rise rapidly upon exposure to a variety of stress factors. GDF15 binds to and activates a heterodimeric receptor, GDNF-family receptor α-like (GFRAL)-RET specifically expressed in the brainstem. The central effects associated with GDF15 include reduction of food intake, emesis, weight loss, delayed gastric emptying, conditioned aversion, and can be regarded as consistent with reactions to various stressors and aversive signals, including the exposure to chemical threats. The rise of circulating GDF15 levels caused by metformin, was associated with an increase of GDF15 expression and release from intestinal cells, and was suggested to be consequent to the metformin-induced mitochondrial dysfunction in these cells [113].

In agreement with its role in transmitting stress signals to the CNS, GDF15 was recently found to activate the protective hypothalamus-pituitary-adrenals (HPA) axis response to specific toxins in mice [114]. Since metformin can increase circulating GDF15 levels, a metformin-induced activation of the HPA axis in a GDF15-dependent manner, may be expected. Actually, based on the GDF15 concentration values associated with metformin administration and with the HPA axis response to specific toxins, respectively [109,113,114], we may conclude that the rise of circulating GDF15 levels upon metformin treatment does not reach the peak value required for the HPA axis activation. To this end, to our knowledge, there is no evidence of such an effect in humans or in experimental animal models.

Finally, it is worth remarking that metformin has obtained the strongest evidence along with GLP-1 agonists and SGLT2 inhibitors for a beneficial effect on the endothelial function, among the available drugs for type 2 diabetes treatment. Targeting the endothelial dysfunction may contribute to prevent or slow-down macrovascular disease associated with diabetes. Although the achievement of glycemic control and the improvement of metabolic health can lead to a reduction of cardiovascular risk factors, there is also evidence that metformin exerts direct effects on endothelial function. A review of this topic is beyond the aim of this work. Moreover, the metformin impact on endothelium and the related cellular and biochemical mechanisms, as suggested by the latest preclinical and clinical investigations, have been comprehensively discussed elsewhere [115].

### 4.4. Possible Mechanisms Underlying the Effects of Metformin in Patients with PCOS

The mechanism by which a treatment with metformin can contribute to the reversal of the clinical manifestations of PCOS, include direct and indirect actions at different levels of the hypothalamic–pituitary–gonadal axis, and the metformin-induced effects on cellular bioenergetics may have a role [47].

In PCOS, the gonadotropin-releasing hormone (GnRH) pulse frequency is increased, which favors increased luteinizing hormone (LH) secretion over that of follicle-stimulating hormone (FSH). Under control of high pulsatile release of LH, the ovary theca cells increase their steroidogenic activity, thereby producing androgens. A relative deficit in FSH secretion drives an impaired follicular development and a reduced aromatase activity, thereby resulting in ovarian follicular atresia, excess androgen accumulation and hyperandrogenemia. In addition, insulin resistance and the compensatory increase in plasma insulin levels progressively worsen this hyperandrogenemia, acting on different tissues. Insulin resistance may indirectly increase the LH pulse amplitude. Moreover, insulin can directly augment the ovarian steroidogenesis and reduce the hepatic expression of sex hormone binding globulin (SHBG), thus increasing the free testosterone levels [43,44,45].

Decreasing hyperinsulinemia with metformin administration can lead to a reduction of circulating androgen levels, an increase of SHBG levels, and inhibition of premature luteinization. Moreover, there is evidence that metformin can directly affect the steroidogenic activity in the ovarian cells, acting through AMPK-dependent signaling pathways [42,44,45,46,47,48,50].

Metformin also contributes to the regulation of gonadotropin secretion and fertility through AMPK-mediated actions at the hypothalamic level and in pituitary gonadotroph cells, which have been recently reviewed [19,47].

A recent experimental study in a rat model of PCOS remarked the link between metformin actions on cellular bioenergetics and its therapeutic effects. The treatment with metformin combined with cyproterone acetate and ethinyl estradiol, reversed the decrease in granulosa cell proliferation and the increase in granulosa cell apoptosis observed in the ovarian tissue of PCOS rats. Moreover, a downregulation of pyruvate kinase (PKM2) and lactate dehydrogenase (LDH-A) expression, was observed in PCOS rat ovaries. The combined pharmacological treatment increased the expression of PKM2 and LDH-A and the lactate and ATP levels, without altering the NAD^+^/NADH ratio, thus suggesting that the reversal of some pathological changes in the ovarian tissue of PCOS rats as observed after the combined treatment, may be associated with the action of metformin on glucose metabolism [46].

Finally, apart from restoring the ovulation cycle, metformin was suggested to improve some endometrial abnormalities during the implantation period, thus increasing the rate of successful pregnancy. In this context, Ohara and coworkers showed that metformin treatment resulted in decreased androgen receptor (AR) expression and increased expression of Homeobox A10 (HOXA10), a transcription factor required for the implantation of embryos, in the endometrium of women with PCOS. This action can be linked to the amelioration of insulin resistance and hyperandrogenism. The authors also performed in vitro experiments, which suggested a direct effect of metformin on endometrial cells, both epithelial and stromal cells. Metformin was able to counteract, at least partially, the upregulation of AR expression and the downregulation of HOXA10 expression in endometrial cell lines exposed to androgen excess [116]. Finally, it is worth adding that the authors themselves remarked some limitations of their study related to the small number of patients, the heterogeneity of reproductive, metabolic and endocrine conditions associated with PCOS, and the use of cell lines incubated with testosterone for in vitro studies in place of primary cell cultures. Their study may suggest further extensive research to better characterize the metformin effects on endometrium in PCOS [116].

### 4.5. Pharmacogenomic Studies for Metformin Action

Despite metformin being the first-line agent for the treatment of type 2 diabetes, metformin monotherapy is not effective in everyone. Moreover, its side-effects may be more marked or intolerable in some patients. causing discontinuation of the treatment. Predicting the individual clinical response to metformin would help to improve the management of type 2 diabetes according to a “precision medicine” approach. Actually, the lack of a well-defined, specific molecular target of metformin mediating its therapeutic effects, and the multiple molecular mechanisms and various target-tissues claimed to be involved in metformin actions, do not help to find a phenotype that can predict the response and tolerance to metformin [117].

Pharmacogenomic studies can be used to categorize patients into subgroups based on their clinical response to the drug and hence to identify genetic and epigenetic markers associated with metformin response. At the same time, they can help to identify genes that encode drug targets, and to gain new insights into the mechanism of action. Before the advent of genome-wide association studies approaches, pharmacogenetic studies of metformin investigated candidate genes, which include genes encoding factors involved in metformin absorption, distribution, and elimination. In this context, the gene *SLC22A1* encodes the OCT1 transporter, which plays a main role in metformin transport into hepatocytes. *SLC22A1* is highly polymorphic, with coding missense single nucleotide polymorphisms (SNPs) affecting its activity [118]. Evidence has been accumulated that OCT1 coding missense polymorphic variants may be linked to reduced response and tolerance to metformin [117]. Genome-wide association studies reviewed by Florez in 2017 [117] showed that common SNP variants detected near the gene encoding the ataxia-telangiectasia mutated kinase (ATM) were associated with glycemic response to metformin in type 2 diabetes. In this context, it is worth remarking that ATM is an upstream kinase linked to the AMPK activation pathway [119]. A significant association was also observed at an SNP in an intron of the glucose transporter GLUT2 (*SLC2A2* gene), which is involved in glucose transport in hepatocytes [120]. Actually, according to the conclusion drawn by the review author, the combined impact of both loci on metformin response was shown to be minimal and other genetic determinants are most likely to be involved [117].

A role of the ATM gene in the hepatic response to metformin also emerged from a genome-wide characterization of differentially expressed genes and gene regulatory elements affected by metformin in human hepatocytes. Among several elements activated by metformin treatment, the authors suggested an enhancer in an intron of the ATM gene, which could be regulating the expression of ATM and two neighboring genes. In the same study, metformin upregulated the activating transcription factor-3 (ATF3) in an AMPK-dependent manner, and this factor could have a significant role in gluconeogenesis repression [121].

More recently, a genome-wide association study searched for epigenetic markers associated with metformin response or intolerance in drug-naïve patients with type-2 diabetes from ongoing prospective studies. The authors analyzed DNA methylation in blood samples and identified 11 sites related to metformin response and 4 sites related to metformin side-effects. The greater the methylation of these sites, the greater was the risk of reduced response to the drug or intolerance to the drug, respectively. The authors remarked that the methylation pattern of these sites in blood met the methylation pattern in adipose tissue, suggesting that the identified epigenetic changes might also regard metabolically relevant tissues for type 2 diabetes. Finally, the authors showed that silencing five genes annotated to the identified sites significantly altered the expression of metformin transporter genes and genes coding for gluconeogenetic enzymes in cultured hepatocytes, further supporting a link to the pharmacology of metformin [122].

On the other hand, a genome-wide analysis of DNA methylation profiles in white blood cells of healthy volunteers treated with metformin at therapeutic doses showed that metformin by itself can induce changes of DNA methylation in short term, thus suggesting that epigenetic regulation may be involved in mediating some metformin actions. Briefly, the research revealed several differentially methylated regions as novel potential epigenetic targets of metformin, and eleven regions with the most consistent changes in the DNA methylation profile were selected. The genes associated with these regions were functionally linked to the regulation of energy metabolism or tumorigenesis. Among them, it is worth pointing out the *CAMKK1* gene, which encodes an isoform of Ca^2+^/calmodulin-dependent protein kinase kinase. CAMKK1 is an upstream kinase phosphorylating AMPK and it is also believed to mediate AMPK-independent glucose uptake in skeletal muscle cells [123].

## 5. The Role of Pyruvate Metabolism in Metformin Actions

Although metformin can affect multiple metabolic- and nonmetabolic pathways in a tissue-specific manner, the previously summarized actions on glucose metabolism suggest further discussing the possible link between pyruvate metabolism and metformin actions in different physiopathological conditions.

**Figure 1:**
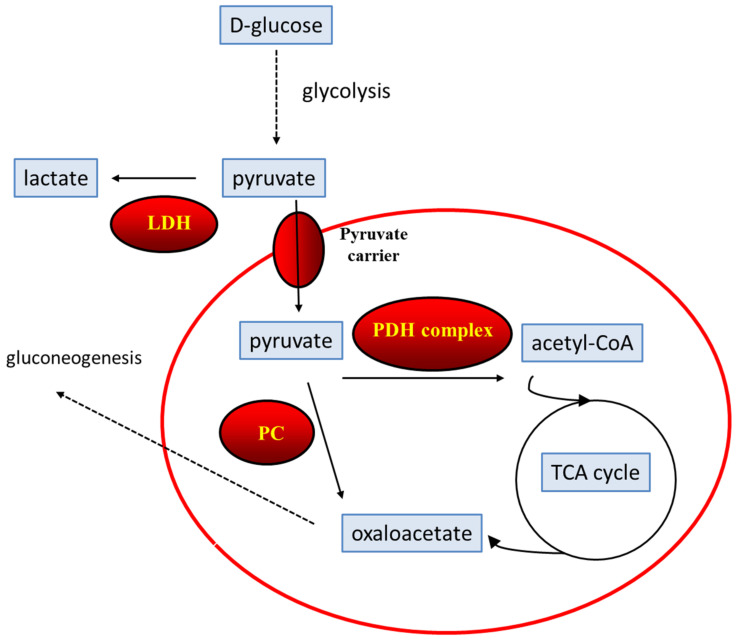
Pyruvate is the product of the glycolytic pathways. Within mitochondria, pyruvate can be oxidized to acetyl-CoA by the pyruvate dehydrogenase (PDH) complex. Alternatively, it can be directed to an anaplerotic reaction catalyzed by pyruvate carboxylase (PC), which generates oxaloacetate. Finally, pyruvate can also be reduced to lactate in the cytosol by lactate dehydrogenase (LDH), This reaction ensures NAD recycling when cells need to maintain a high glycolytic rate.

Pyruvate is an intermediate at an important metabolic branch point affecting both ATP production and cytosolic/mitochondrial redox state. In summary, pyruvate can be oxidized to acetyl-CoA by the pyruvate dehydrogenase (PDH) complex. Acetyl-CoA enters the tricarboxylic acid (TCA) cycle and is further oxidized. The resulting reducing equivalents NADH and FADH_2_ are ultimately used to carry out complex I-dependent cellular respiration, which leads to the synthesis of ATP molecules. Alternatively, inside mitochondria, pyruvate can be directed towards an anaplerotic reaction catalyzed by pyruvate carboxylase (PC) which generates oxaloacetate, and supplies carbon for gluconeogenesis. Finally, instead of being directed to mitochondria, pyruvate can also be reduced to lactate in the cytosol by lactate dehydrogenase (LDH) (Figure 1). This reaction ensures NAD recycling when cells need to maintain a high glycolytic rate [124,125,126,127,128].

### 5.1. The PDH Complex Activity: Regulatory Mechanisms and Consequences of Congenital or Acquired Deficiency

PDH is a key rate-limiting enzyme that determines the metabolic balance between glycolysis vs. mitochondrial oxidative phosphorylation, and the oxidative removal of glucose, pyruvate and metabolites in equilibrium with pyruvate (alanine and lactate). PDH is an enzymatic complex formed by different subunits. Its activity is regulated allosterically and by reversible phosphorylation at three specific sites of the PDHE1α subunit in response to the availability of substrates and end-products. PDH activity is inhibited by the phosphorylation of the PDHE1α subunit mediated by four pyruvate dehydrogenase kinases (PDKs). In fact, phosphorylation at any site, including serine-293, leads to the inhibition of the complex in vitro [124,125,128].

The acute control of the PDH complex activity is mediated by end products (acetyl-CoA, NADH, and intramitochondrial ATP) which increase the activity of PDKs, leading to PDHE1α phosphorylation. On the other hand, pyruvate can inhibit the PDK activity, thus leading to the removal of the block. Some halogenated compounds showing structural similarity to pyruvate, cause the same effect as pyruvate. To this purpose, dichloroacetate (DCA) is a PDK inhibitor extensively used in preclinical and clinical investigations. Finally, the PDH phosphatase (PDPs) activity is positively regulated by insulin and by magnesium and calcium ions [128,129].

The association between mitochondrial dysfunction leading to accumulation of reactive oxygen species (ROS) and pathophysiology of aging and age-related disorders has long been considered. The PDH complex is central to mitochondrial activity and has been suggested as a potential therapeutic target for age-associated conditions including reduced glucose tolerance, obesity-related sarcopenia, neurodegenerative diseases, and cancer. In detail, a similarity between some clinical features of congenital PDH complex deficiency [130] and some disorders associated with aging has been remarked. In fact, the biochemical characterization of the congenital deficiency status has contributed to provide insight into the mechanisms underlying age-related disorders [129].

Congenital PDH complex deficiency has been associated with aerobic glycolysis and increased lactate production. In this context, glucose carbon is diverted from acetyl-CoA synthesis and used for oxaloacetate synthesis through pyruvate carboxylase (PC). The overall flux through the TCA cycle is reduced [131]. Finally, in skin fibroblasts from patients harboring mutations of the PDHE1α subunit, the hypoxia inhibitory factor 1α (HIF1α) was found to be overexpressed. This factor transactivates numerous genes involved in cell metabolism, growth, and survival, including glycolytic enzymes and PDKs [132]. The HIF1α-mediated transactivation of PDKs contributes to further downregulate PDH activity and oxidative phosphorylation [133,134,135]. Moreover, glycolytic products such as pyruvate can stabilize HIF1α by inhibiting its degradation in the cytoplasm, thus triggering a positive feedback loop whereby the HIF1α overexpression is maintained by the increased glycolytic flux and, in turn, contributes to further decreasing the residual PDH complex activity [129,135,136].

The pathophysiological decrease of the PDH complex activity through normal aging or acquired diseases, displays similar biochemical concomitants with respect to the congenital deficiency [129] (Table 3).

In aging, an increase of long-chain fatty acid (LCFA) beta-oxidation in skeletal muscle may contribute disproportionately to TCA flux and oxidative phosphorylation. The consequent raise in acetyl-CoA, NADH, and ultimately ATP levels increases the PDK activity, thereby inhibiting the PDH complex activity. This remodeling contributes to the age-associated decline in the skeletal- and cardiac muscle response to insulin. The PDH complex inhibition also increases the reduction of pyruvate to lactate, which is carried out of cells by two H^+^⁄ lactate monocarboxylate transporters, MCT1 and -4, the latter being regulated by HIF1α. Moreover, a decreased insulin action at peripheral tissues removes the insulin-mediated tonic stimulation of PDPs, which helps to maintain PDHE1α in its unphosphorylated-, catalytically active form, thereby contributing to the inhibition of the PDH complex activity [129,137,138].

In cancer cells, the upregulation and stabilization of HIF1α contributes to the Warburg effect. HIF1α transactivates genes related to the increase of glucose uptake, glycolysis, and lactate production, including PDKs, and causes cancer cells to rely on glycolysis for energy, rather than on oxidative metabolism [83]. The reduction in oxidative phosphorylation decreases superoxide production by the respiratory chain, resulting in the inhibition of pro-apoptotic signaling pathways. In addition, the MCT1 and MCT4-mediated extrusion of lactate and protons, reduces tumor acidosis, thereby promoting tumor survival [129,137,138,139,140].

Other molecular mechanisms cooperate to the PDH complex inhibition in cancer cells, in addition to factors regulating the PDHE1α phosphorylation. In this regard, sirtuin-4 (SIRT4) was shown to possess lipoamidase activity and the removal of lipoamide cofactors from the E2 component, resulting in downregulation of the PDH complex activity [141]. The SIRT4 lipoamidase activity was found to be induced by glutamine, which is an important energy substrate involved in metabolic remodeling in cancer cells and other proliferative conditions [128,142,143]. These findings significantly contribute to further characterize the role played by intramitochondrial sirtuins (SIRT3-5) in regulating cellular energetics, signaling, and apoptosis [144].

The consequences of congenital or acquired deficiency in PDH complex activity, confer significance to how this perturbation may be therapeutically reversed. To this purpose, DCA was shown to reduce the PDK activity in skin fibroblasts from patients with genetic deficiency of PDHE1α as well as in tumor cells, thus reactivating the PDH complex activity and causing the reversal of the metabolic balance between glycolysis and oxidative phosphorylation [128,129,131,145].

### 5.2. Metformin Impact on Pyruvate Metabolism

Metformin can affect the pyruvate metabolism in a tissue-specific and concentration-dependent manner in both normal and tumor cells. Metformin can modulate enzymatic activities in metabolic pathways by altering the cellular redox balance and the levels of substrates, metabolic intermediates, and allosteric enzymatic effectors, regardless of changes in enzyme expression [7,8,12,39]. Actually, metformin actions may also include the induction of transcriptomic changes, mimicking those raised by caloric restriction [146].

Some metabolic rearrangements associated with the response to high metformin concentrations within cells may resemble a deficiency of the PDH complex activity, with a decrease in TCA cycle flux and mitochondrial NADH oxidation, the enhancement of glycolysis and lactate production, and the switch to glutamine utilization to provide ATP or biosynthetic intermediates [12,147].

Focusing on the induction of transcriptomic changes, a crossover study in elderly humans with reduced glucose tolerance provided evidence that a prolonged treatment with metformin can exert tissue-specific effects on gene expression in skeletal muscle and subcutaneous adipose tissue. In skeletal muscle, pyruvate metabolism, PDH complex regulation, glycolysis, and gluconeogenesis along with DNA repair systems were the overrepresented pathways in terms of changes in gene expression caused by metformin vs. placebo. Among the transcriptomic changes that were suggested to be relevant to any healthy aging-promoting effect, it is worth remarking the upregulation of *PCK1* in skeletal muscle. This gene encodes for phosphoenolpyruvate carboxykinase (PEPCK) [148,149].

PEPCK is a well-known key enzyme in the gluconeogenetic pathway in liver and kidney cortex. Actually, *PCK1* is expressed in various tissues, including skeletal muscle, and its metabolic role may be different in distinct tissues. In liver and adipose tissue, it is involved in glyceroneogenesis with the synthesis of glycerol-3-phosphate from precursors other than glucose and glycerol [148].

The overexpression of the cytosolic form of PEPCK in transgenic mice was related to enhanced levels of physical activity which extended into old age, along with an increased number of mitochondria and increased oxidative capacity, a high concentration of triglycerides in skeletal muscle cells and the preferential use of triglycerides vs. glucose as energy source [148]. Since metformin treatment is believed to enhance the *PCK1* gene expression in skeletal muscle [149], the article by Hakimi and coworkers suggests that PEPCK activity can contribute to repattern cell bioenergetics and to induce metabolic flexibility in skeletal muscle cells exposed to metformin. Actually, the role of cytosolic PEPCK in skeletal muscle, is not immediately apparent. PEPCK activity was shown to increase in response to exercise in rats, and to decrease rapidly after the end of exercise [150]. While they discussed the effects of its overexpression in mice, Hakimi and coworkers suggested some possible functions of cytosolic PEPCK in skeletal muscle cells. Briefly, cytosolic PEPCK is a reversible enzyme in vitro; therefore, its activity may contribute to both the generation (anaplerosis) and the removal (cataplerosis) of TCA cycle intermediates when a major increase in TCA cycle flux occurs during exercise [148]. Cataplerosis is especially important when protein turnover generates considerable amounts of free amino acids, with subsequent oxidation [151,152]. A second possible role of cytosolic PEPCK is glyceroneogenesis, although the occurrence of glyceroneogenesis in skeletal muscle cells is in dispute. Finally, an excess of cytosolic PEPCK activity in brown adipose tissue and skeletal muscle has been suggested to support a cycle between cytosol and mitochondria which converts mitochondrial GTP to ATP by enhancing the oxidation of acetyl units. Actually, such a cycle would depend on an efficient exchange of nucleotides between cytosol and mitochondria [148,153,154].

Finally, metformin may affect the PDH complex activity at a post-transcriptional level, by inhibition of OCT1-mediated thiamine uptake in the intestine and other tissues. Indeed, the active metabolite thiamine pyrophosphate (TPP) is an essential enzyme cofactor of the PDH complex. Although thiamine transport is also mediated by high-affinity sodium dependent specific transporters (SLC19A2 and 19A3), OCT1 is a high-capacity transporter and is believed to play a major role for thiamine disposition in specific tissues, such as liver and intestine [155,156,157]. In these tissues, metformin may reduce the TPP levels by competitive inhibition of OCT1-mediated thiamine uptake. Indeed, evidence has been provided that metformin can inhibit the OCT1-mediated thiamine uptake in vitro with a 1.4 mM IC_50_ value, and can reduce the plasma levels of thiamine and TPP in vivo in mice. However, in the same in vivo study, no significant effect on the liver thiamine levels was apparent [157].

An effective inhibition of thiamine uptake causing TPP depletion could significantly contribute to metformin actions on cellular energy status and metabolic functions. Indeed, at cellular levels, thiamine depletion would be expected to decrease the pyruvate oxidation to acetyl-CoA and hence the ATP production by Krebs cycle, which in turn would lead to AMPK activation and enhanced fatty acid beta-oxidation. In liver, thiamine depletion may contribute to metformin action on glucose and lipid metabolism, with a decrease of hepatic triglyceride content. Finally, thiamine deficiency may also account for the major, life-threatening side-effect of biguanides such as metformin and phenformin, which is lactate acidosis. In summary, according to preclinical studies [157], metformin treatment can reduce the systemic levels of thiamine, and hence it may facilitate the occurrence of thiamine deficiency in at-risk populations. However, the affinity of OCT1 for thiamine and metformin has been shown to vary significantly between species. Since the affinity of human OCT1 for both compounds is lower compared with mouse OCT1, the contribution of human OCT1 to the cellular uptake of thiamine and especially of metformin, may be much lower than that of mouse OCT1. Hence, the use of rodent cells for predicting OCT1-related tissue distribution of metformin and possible pharmacokinetics interactions between metformin and thiamine in humans may reveal to be misleading [158].

Further evidence supporting a possible interaction between metformin administration and thiamine uptake in the intestine has been provided in recent years. Metformin was shown to be also an inhibitor of thiamine transporter 2 (SLC19A3) and the inhibition kinetics analysis led to the conclusion that metformin can reach intestinal concentrations that may result in SLC19A3-mediated interaction with thiamine uptake. In summary, despite metformin administration not being related to a clear thiamine deficiency in patients, a decrease of systemic thiamine laboratory values is possible, particularly in at-risk populations (e.g., individuals with alcoholism) [159].

## 6. Metabolic Rearrangements That Occur upon Metformin Treatment in Normal and Tumor Cells

The characterization of metformin actions on cell metabolism have helped to elucidate the mechanisms underlying the pharmacological activity of metformin in different physiopathological conditions, and also to determine the metabolic profiles associated with the sensitivity or resistance of distinct tumor cell types to metformin. In this regard, it is important to outline the compensatory responses and metabolic adaptations engaged by distinct proliferating cell populations, both normal and tumor cells, in response to metformin.

It is worth remarking that varying metformin concentrations in vitro may significantly affect its impact on cell metabolism [59,60]. In detail, there still remains some debate regarding a central role of the mitochondria respiratory chain in mediating the metformin effects in various cell types [12,34,38,60]. Nevertheless, when used at concentrations that are effective at inhibiting the mitochondrial complex I, metformin is generally believed to cause a transient decrease in cellular energy status associated with a decrease in NADH oxidation and TCA flux, and low levels of TCA metabolites. The malate-aspartate shuttle runs in the opposite direction compared to what happens in cells not exposed to metformin: NADH is exported to the cytosol in the form of malate, which is converted into oxaloacetate, which is in turn transaminated into aspartate by cytosolic AST. In proliferating cells, all these effects can elicit a cytostatic state with reduced proliferation. However, if the compensatory responses are not enough to achieve a new energy balance, proliferating cells may undergo apoptotic death [7,12].

A summary of the compensatory changes conferring metabolic flexibility to metformin-treated cells may include the activation of AMPK in order to stimulate catabolic pathways and to inhibit anabolic activities, the enhancement of glycolysis and the switch to glutamine utilization to provide ATP or biosynthetic intermediates [12,147]. In this context, L-glutamine can undergo either oxidative or reductive metabolism within cells. In detail, glutaminase catalyzes the conversion of L-glutamine to L-glutamate, which is then reduced to alpha-ketoglutarate, a TCA cycle intermediate, by glutamate dehydrogenase. The reductive metabolism may lead to the replenishment of the TCA cycle intermediates (anaplerosis) by generating citrate from alpha-ketoglutarate, with a decrease in ATP production. Alternatively, when anaplerosis is not required, the oxidative metabolism of L-glutamine contributes to the ATP production [160].

After prolonged exposure to metformin, the adaptive response to the metabolic stress condition is mediated by the increased expression of enzymes involved in rearranging and rerouting metabolic flux, and the upregulation of the metabolic regulator PGC-1α is thought to play a key role [12].

PGC-1α is a key regulator of oxidative phosphorylation (oxphos) and mitochondrial biogenesis. Actually, PGC-1α has other functions apart from its role in mitochondrial metabolism, and can support various metabolic programs in cancer cells [161,162]. More in detail, in cancer cells exposed to metformin, the upregulation of PGC-1α has been argued to reprogram cell metabolism by promoting an alternate source of ATP production through the enhancement of glycolysis, and diverting mitochondrial metabolites that would normally be used for ATP production, for use in anabolic reactions. In summary, after prolonged exposure of cancer cells to metformin, the increase in PGC-1α levels may enhance their metabolic flexibility, thus contributing to the onset of drug resistance [12,163,164,165].

PGC-1α activity may be also regulated by means of post-translational modifications, which include phosphorylation by AMPK and de-acetylation by sirtuin-1 (SIRT1), a member of the class III (NAD+-dependent) histone deacetylases (HDACs) and a metabolic sensor of the NAD^+^/NADH redox state. Both modifications enhance the PGC-1α activity [53,166,167]. Metformin via AMPK increases the activity of SIRT1, resulting in the deacetylation of downstream SIRT1 targets [3,168]. Actually, cancer cell-specific post-translational modifications on PGC-1α have not been well characterized yet.

### Metabolic Rearrangements in Pituitary Tumor Cells Compared with Normal Proliferating Cells

In the last decade, in vitro studies evidenced that metformin can reduce the growth and viability of pituitary tumor cells, acting through AMPK-dependent and AMPK–independent mechanisms. Such an effect has been seen on multiple pituitary tumor cells lines [169,170,171,172,173]. Actually, more contradictory results have emerged from studies on primary cultures from human pituitary adenomas and no clear evidence supporting an efficacy of metformin as an adjuvant therapy for prolactin- or GH-secreting tumors, has emerged from clinical data [19,35]. This topic is extensively reviewed in the last section. Further pondering the results of the available preclinical investigations may help to understand which intrinsic features may contribute to make distinct pituitary tumor cell populations more responsive, less responsive, or fully resistant to the antitumor activity of metformin, and hence to consider the opportunity of new preclinical and clinical investigations on selected pituitary malignancies.

Various studies stressed the metformin-induced changes in the activity of signaling pathways regulating pituitary tumor cell growth and viability [19,35,170,171]. More recently, our research group tried to explore the impact of metformin on the metabolic activity of pituitary tumor cells. More precisely, we compared rat pituitary tumor cell lines responsive to metformin to rat myogenic precursors as a model of rapidly proliferating, undifferentiated, normal cells whose growth was not negatively affected by metformin. In the attempt to evidence any difference in the compensatory metabolic response to metformin between pituitary tumor cells and normal cells, we ruled out the chance to compare the pituitary tumor cells to normal pituitary cells because the proliferation rate is related to the ATP requirement by cells, with rapidly proliferating cells requiring more ATP than differentiated, non-proliferating cells [12,20].

Our study [20] provided evidence that metformin can exert differential effects on redox activity and energy formation in pituitary tumor cells compared to normal proliferating cells, with consequences on their growth in vitro. Moreover, it also suggested that the pyruvate metabolic branch point is most likely to play a main role in the variability of the cell response to metformin. The study was mainly based on the analysis of two markers, the reduction of the tetrazolium salt MTT within cells [174] and the ATP content in whole cell extracts. We used metformin concentrations within the 10(−4)–10(−3) M range, which means at concentrations that are expected to be active on the mitochondrial respiratory chain [12,38,60]. After the treatment with metformin or its vehicle, cells were incubated in buffered balanced salt solution supplemented with distinct metabolic substrates for short time intervals, before performing the final assays.

**Figure 2:**
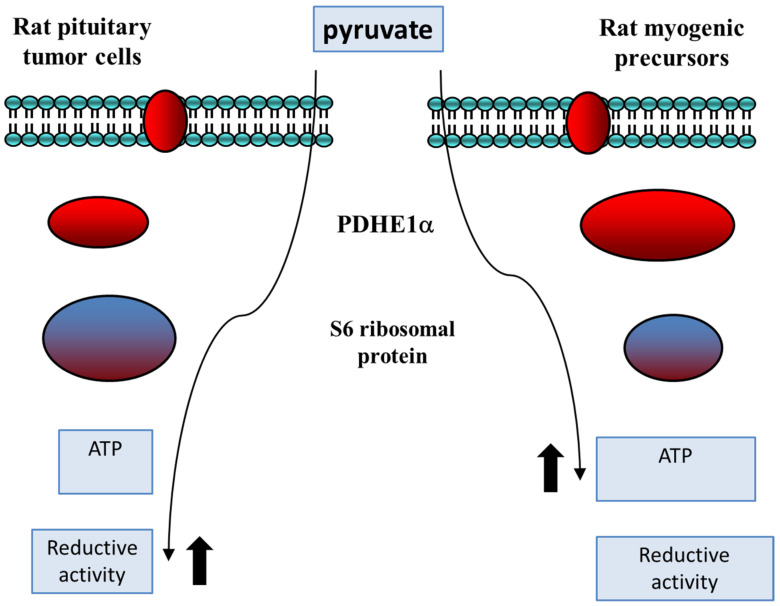
Metformin decreased the growth and viability of rat pituitary tumor cells in vitro, whereas it did not affect the growth of rat myogenic precursors, which are normal, undifferentiated, and rapidly proliferating cells. Rat pituitary tumor cells and rat myogenic precursors differed significantly in some features of their metabolic profile in basal culture conditions: the reductive activity (reduction of the tetrazolium salt MTT within cells) and the ATP content were lower in pituitary tumor cells vs. myogenic precursors. The two cell types also differed significantly in the PDH complex expression levels (PDHE1α protein level), the S6 ribosomal protein levels, and their response to specific metabolic substrates. In this context, short incubations with pyruvate caused an increase of ATP content in myoblasts, without altering the reductive activity. On the other hand, in rat pituitary tumor cells, pyruvate enhanced the sole reductive activity.

Rat pituitary tumor cells and rat myogenic precursors, herein called myoblasts, differed significantly in some features of their metabolic profile in basal culture conditions. The reductive activity and the ATP content were lower in pituitary tumor cells vs. myoblasts, due to either a lower production or a higher consumption of reducing equivalents and ATP. The two cell types also differed significantly in the PDH complex expression levels. Myoblasts showed higher PDHE1α expression, which may be regarded as an index of greater size of the mitochondrial system or, at least, greater capacity to direct pyruvate to mitochondrial oxidation. On the other hand, rat pituitary tumor cells showed higher levels of S6 ribosomal protein, which suggest a substantial protein synthesis activity. The analysis of the cell response to a specific metabolic substrate, in detail glucose or pyruvate, confirmed a significant difference at the level of the pyruvate metabolic branch point between the two cell types. In myoblasts, the increase of ATP levels in response to pyruvate, in the absence of any change in the reductive activity, suggested an efficient pyruvate decarboxylation to acetyl-CoA and augmented mitochondrial energy formation. On the other hand, in pituitary tumor cells, the incubation with pyruvate enhanced the sole reductive activity without increasing the ATP production, in agreement with a less efficient conversion of reducing equivalents to energy equivalents in the mitochondrial electron transport chain (Figure 2).

The increased reductive activity also ruled out a prompt conversion of pyruvate to lactate in these cells. This reaction would have consumed NADH with a negative impact on the cell reductive activity [20]. Alternatively, the selective impact of pyruvate on the reductive activity in rat pituitary tumor cells, may also be explained by the use of pyruvate in the anaplerotic reaction catalyzed by pyruvate carboxylase, which generates TCA cycle intermediates and mitochondrial reducing equivalents [148,175]. Finally, the pyruvate excess might have shunted the available intracellular glucose to the pentose phosphate pathway (PPP), which is a source of reducing power for biosynthesis [176].

**Figure 3:**
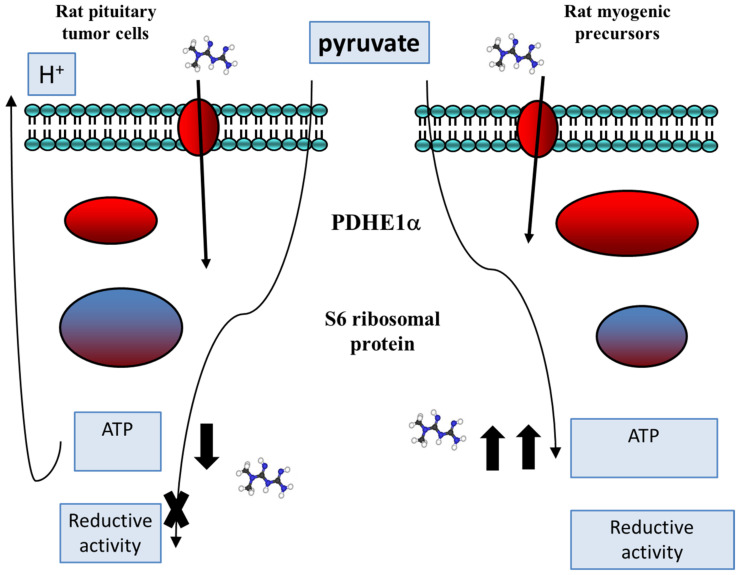
The treatment with metformin negatively affected the ATP production in pituitary tumor cells. Moreover, metformin caused the reversal of the pituitary tumor cell response to pyruvate, as regards the enhancement of the reductive activity (reduction of the tetrazolium salt MTT within cells), and increased the acidification of the extracellular medium. On the other hand, metformin enhanced the energy formation (ATP content) in myogenic precursors, and sustained their growth over prolonged incubations.

The treatment with metformin enhanced the glucose utilization in myoblasts, thus increasing the energy formation and sustaining their growth over prolonged incubations, whereas it negatively affected the ATP production and, to a lower extent, the reductive activity in pituitary tumor cells. Moreover, metformin caused the reversal of the pituitary tumor cell response to pyruvate, as regards the enhancement of the reductive activity. In other terms, metformin further decreased the pyruvate oxidative metabolism or pushed the conversion of pyruvate to lactate. This conclusion was supported by the acidification of the extracellular medium in pituitary tumor cell cultures (Figure 3) [20].

A recent study provided evidence that metformin can enhance lactate production in cancer cells. Indeed, metformin was shown to increase the hyperpolarized [1-13 C]lactate conversion rate in an animal model of breast cancer [83]. The hyperpolarized [1-13 C]lactate production has been suggested as a biomarker for cancer occurrence as well as for monitoring tumor response to therapy. In general, an increase in [1-13 C]lactate conversion rate is regarded as a sign of cancer occurrence or tumor growth, whereas a decrease is related to tumor suppression. Actually, the study by Choi and coworkers suggests that the in vivo administration of metformin may challenge this paradigm. Indeed, metformin revealed to be effective as an adjuvant treatment combined with radiation therapy and increased the tumor suppression effect compared to radiation therapy alone, nevertheless it enhanced the hyperpolarized [1-13 C]lactate production [83].

In pituitary tumor cells exposed to metformin, the comparison between the effects of glucose and the effects of pyruvate supported a prevailing role of the early steps of glucose metabolism, upstream of pyruvate, in the production of reducing equivalents and in the recovery of ATP. Indeed, metformin did not further limit the glucose utilization for reductive activity and energy production by these cells. In agreement with a prevailing role of the cytosolic metabolism of glucose, a synergistic interaction between 2-deoxy-D-glucose (2-DG) and metformin was seen in pituitary tumor cells [20]. 2-DG can be phosphorylated by hexokinase to form 2-deoxy-D-glucose 6-phosphate which cannot be further metabolized and accumulates in the cells where it blocks glycolysis acting as a noncompetitive inhibitor of hexokinase [145,176]. As a consequence, the accumulation of glucose 6-P may shunt D-glucose to the PPP [176]. The synergistic interaction between 2-DG and metformin in pituitary tumor cells also provided further confirmation to the conclusions of previous studies showing that a combination of hypoglycemia and metformin can significantly inhibit tumor cell metabolism and growth [177,178].

In myoblasts, the addition of 2-DG reversed the enhancement of the metabolic activity triggered by metformin, suggesting that it was dependent on the enhancement of the glycolytic pathway. To this end, it is worth remarking that metformin by itself tended to increase the extracellular glucose consumption by myoblasts to produce energy [20]. Our data might seem to be in contrast with previous studies, which have suggested that metformin is able to increase the mitochondrial energy formation in rat myoblasts [70]. Actually, when D-glucose is the only available metabolic substrate and carbon source, any block of glycolysis may be expected to reduce also the production of TCA cycle intermediates. Hence, in our experimental conditions, 2-DG might have affected both a cytosolic and mitochondrial response to metformin in myoblasts.

Metformin is generally believed to cause a transient decrease in NADH oxidation associated with a decrease in TCA flux, when used at concentrations that are effective at inhibiting the mitochondria respiratory chain [12,179]. The quantification of NAD^+^, NADH and their ratio in whole cell extracts, confirmed this effect in rat pituitary tumor cells and rat myoblasts. We observed an increase of NADH levels and a decrease of the NAD^+^/NADH ratio in both cell types, even more pronounced in pituitary tumor cells [20].

In summary, despite the primary action of metformin is most likely the same in rat pituitary tumor cells compared to rat myoblasts, a differential efficacy of the compensatory responses triggered by the treatment, leaded to opposite outcomes on cell metabolic activity and ultimately on cell viability and growth. A substantial difference between the two cell types, which might affect their responses to metformin, was associated with the fate of pyruvate within cells at a metabolic branch point linking various anaplerotic and catabolic pathways. In this context, the PDH complex expression levels may play a central role.

Focusing on the metabolic activity of skeletal muscle cells treated with metformin, the increase of cellular ATP content as observed in rat myoblasts [20,70] found further confirmation in a study with mouse myoblasts. This study in C2C12 cells also evidenced a functional interaction between metformin and miR-378a-3p, a microRNA highly expressed in skeletal muscle, liver and brown adipose tissue, and implicated in the regulation of glucose metabolism and mitochondria activity [180]. When miR378a-3p was overexpressed in mice, it was found to improve the systemic energy homeostasis and to ameliorate obesity by inducing the pyruvate-PEP futile cycle in skeletal muscle and enhancing lipolysis in adipose tissue. The pyruvate-PEP futile cycle has been suggested to increase the energy expenditure in skeletal muscle, and consequently to promote the crosstalk between skeletal muscle and adipose tissue, with an improvement of the body weight homeostasis [181]. At a cellular level, the activation of this futile cycle could account for a decrease of ATP content in myoblasts. In C2C12 cells, the increase of ATP content caused by metformin was abolished when miR378a-3p was blocked [180]. These data suggest the intriguing hypothesis that metformin can increase the ATP content of myoblasts by counteracting the pyruvate–PEP futile cycle activity induced by miR378a-3p, and provide further evidence that the pyruvate metabolic check point plays a major role in cell response to metformin.

The relevance of the PDH complex regulation to the tumor cell response to metformin, is not a new finding. In oral squamous cell carcinoma, metformin reduced HIF-1α expression and increased PDH expression [182]. Moreover, in breast cancer cells, the tumor protein D54 binds to and stabilizes the PDHE1α subunit, blocking its phosphorylation. In these cells, the suppression of D54 was shown to cause PDHE1α degradation, leading to reduced PDH complex activity and increased resistance of breast cancer cells to metformin [183]. Actually, the previously mentioned study in pituitary tumor cells pointed out that an opposite role of PDH in regulating the tumor cell response to metformin is also possible. Indeed, lower levels of PDHE1α expression and lower ATP content in rat pituitary tumor cells compared to rat myoblasts, were associated with higher sensitivity to the inhibitory action of metformin on cell growth and viability [20].

Finally, the compensatory changes conferring metabolic flexibility to metformin-treated cells include the switch to glutamine utilization to provide ATP or biosynthetic intermediates [12,160]. The analysis of the cell response to L-glutamine in rat myoblasts, suggested a prevailing oxidative metabolism of L-glutamine. The response of rat pituitary tumor cells was more complex. L-glutamine did not significantly alter the metabolic activity of these cells upon basal conditions. On the other hand, when the cells had been treated with metformin, the addition of L-glutamine to the incubation medium rescued them from the drop of ATP production caused by metformin [20].

It has been previously reported that metformin increases the dependency of prostate cancer cells on L-glutamine reductive metabolism [160]. In rat pituitary tumor cells, the reversal of the metformin effect on ATP production can be better explained by the enhancement of L-glutamine oxidative metabolism. Anyway, both studies contribute to highlight the interaction between metformin and L-glutamine metabolism in tumor cells, and we may conclude that the switch to L-glutamine utilization may cooperate to pituitary tumor cell escape from metformin treatment.

## 7. Cell Signaling and the Anticancer Activity of Metformin

The potential antitumor activity of metformin is determined by systemic actions and direct actions on cells [3,7,184,185,186,187,188].

Epidemiological evidence has shown the association between type 2 diabetes, insulin resistance, hyperinsulinemia, and cancer [7,189]. At a systemic level, metformin reduces insulin resistance and improves plasma glucose control, thus decreasing hyperinsulinemia, insulin receptor activation in neoplastic cells and pre-neoplastic cells, and glucose concentration in the tumor microenvironment. Moreover, metformin can decrease the expression of proinflammatory cytokines and circulating proinflammatory cytokine levels [7,187,190].

At a cellular level, the effects of metformin have been suggested to resemble the effects of calorie restriction strategies and interventions on energy balance, possibly leading to the same claimed health benefits, which include a decrease of aging-associated inflammation, reduced risk of developing cancer and extended lifespan. In calorie restriction, the energetic constraints trigger an increase in AMPK signaling and a decrease in mammalian target of rapamycin complex 1 (mTORC1) signaling. These effects are believed to induce reallocation of cellular resources from growth/proliferation to somatic maintenance, i.e., proteostasis and maintenance of mitochondrial protein turnover, and are related to autophagy induction [19,23,191,192,193,194]. Autophagy is a self-degradation process recycling cellular components, and contributes to cellular energy homeostasis through degradation of dysfunctional mitochondria (mitophagy) [15]. Metformin is believed to induce the same signaling changes caused by calorie restriction [23,191].

The metformin effects related to its antitumor activity are mediated by AMPK-dependent and AMPK-independent signaling pathways, and include non-site-specific effects such as decreased production of biosynthetic precursors derived from TCA cycle and lipogenesis, and altered production of reactive oxygen species (ROS) [3,7]. As to the latter effect, more precisely, metformin was shown to either induce or to inhibit ROS production in different cell populations, acting through mechanisms that are still partially understood [7,71,195,196].

The indirect AMPK activation caused by metformin leads to the mTORC1 signaling pathway inhibition and inhibition of lipogenesis. More in detail, the AMPK-dependent actions of metformin include: mTORC1 inhibition, regulation of fatty acid synthesis, which is necessary for the assembly of cellular membranes, and regulation of cell metabolism [19,197,198].

Apart from directly targeting a number of metabolic enzymes and transporters, such as glucose transporters GLUT1 and GLUT4, glycogen synthase (GS), acetyl-CoA carboxylase (ACC), and hydroxymethylglutaryl-CoA reductase (HMGCR), AMPK also regulates cell metabolism at the transcriptional level by phosphorylating sterol regulatory element-binding protein 1 (SREBP1), ChREBP, PGC-1α, and transcription factor forkhead box O3 (FOXO3) [7,119,120,121,122,123,124,125,126,127,128,129,130,131,132,133,134,135,136,137,138,139,140,141,142,143,144,145,146,147,148,149,150,151,152,153,154,155,156,157,158,159,160,161,162,163,164,165,166,167,168,169,170,171,172,173,174,175,176,177,178,179,180,181,182,183,184,185,186,187,188,189,190,191,192,193,194,195,196,197,198,199,200,201]. In this context, metformin was also shown to reduce the signal transducer and activator of transcription-3 (STAT3) phosphorylation at ser-727 and tyr-705 in triple negative breast cancer (TNBC) cell lines, AMPK-dependently [202]. The Janus kinase (JAK)-STAT3 signaling pathway plays a role in tumor cell metabolic reprogramming, and cooperates with the HIF-1α overexpression to shift cell metabolism towards aerobic glycolysis [203,204]. In summary, in contrast with the role of AMPK activation in mediating the compensatory changes conferring metabolic flexibility to alleviate the energetic stress associated with metformin [12,147], activated AMPK can also promote the toxicity of metformin in tumor cells, acting in a cell-context dependent manner [202,203,204].

The inhibition of STAT3-mediated signaling by metformin, has been confirmed in various experimental models [170,190,204,205,206], including a transgenic mouse model of follicular thyroid cancer [16]. In these mice, the overactivation of the leptin-JAK2-STAT3 signaling pathway caused a more aggressive tumor progression. More in detail, the intake of a high fat diet drove the development of obesity and elevated the serum leptin levels. These metabolic alterations were associated with increased tumor cell proliferation, vascular invasiveness, occurrence of anaplasia, and shortened survival compared to lean littermates. In mice fed a high-fat diet, metformin reduced the activity of the STAT3-ERK-vimentin and fibronectin-integrin signaling pathways, with the outcome of blocking cancer cell vascular invasion and anaplasia and delaying tumor progression. Actually, metformin did not significantly reduce protein synthesis, lipid metabolism, or ultimately tumor growth in both obese and lean mice. Moreover, no AMPK-mediated inhibition of the mTOR-p70S6K pathway was observed in the tumor tissue. Since the PI3K-AKT signaling pathway was found to be highly activated in the thyroid of these transgenic mice, the authors concluded that the enhanced AKT activity might have overwhelmed the AMPK-mediated action of metformin in the tumor cells. Again, this study supports the conclusion that metformin actions in tumor cells, are dependent on cellular context and pre-existing alterations of signaling pathways [16].

The functional antagonism between metformin and PI3K-AKT-activating stimuli, also involves the regulation of the transcription factor FOXO3, which in turn modulates autophagy-related genes [204,207]. Indeed, FOXO3 is a target of AMPK, and the AMPK-mediated FOXO3 phosphorylation increases its nuclear localization and transcriptional activity [208]. By activating AMPK, metformin is able to contrast the PI3K-AKT-derived signals, which act oppositely and promote FOXO3 degradation [209].

Focusing on the interaction between the AMPK signaling pathway and the mTORC1 pathway, therapeutic concentrations of metformin can promote the formation of a signaling complex on the lysosomal surface with the ability to inversely regulate the two pathways at the same time, resulting in significant consequences in terms of autophagy machinery regulation [7,210].

The recruitment and activation of AMPK at the lysosomal surface caused by metformin leads to a direct mTORC1 inhibition through Raptor phosphorylation and tuberous sclerosis complex (TSC) phosphorylation [7,211,212]. In detail, AMPK phosphorylates TSC2 on amino acid residues which are distinct from those targeted by growth factor pathways, resulting in TSC2 activation. Then, by downregulating the Rheb factor, which directly binds and activates mTORC1, TSC2 inhibits mTORC1. In addition, the phosphorylation of the subunit Raptor contributes to mTORC1 inhibition.

Metformin can also regulate the mTORC1 activity in AMPK-independent manner. In detail, in the signaling complex associated with lysosomes, Ragulator and vATPase factors act as guanine exchange factors and convert RagA and RagC to their nucleotide-bound active state (RagA-GTP and RagC-GDP, respectively), which is competent for the mTORC1 activation through the interaction with Raptor. Actually, in an alternative pathway, which involves the nuclear pore complex, RagC acquires GDP and hence becomes competent for the mTORC1 activation in the nucleus. The ATP depletion related to metformin action as observed in some cell types can alter the function of the nuclear pore complex, resulting in nuclear exclusion of RagC and decreased mTORC1 activation [7,213].

In the last decade, metformin has been shown to target Sonic Hedgehog (Shh) signaling in various tumor types, and this action can cooperate to its antitumor activity. The Shh signaling pathway regulates cell growth, survival, and differentiation during embryonic and postnatal development, but it can be found re-activated in several tumors. Briefly, binding of the Shh ligand to its cell surface receptor Patched (Ptch) leads to the de-repression of the transmembrane repressor Smoothened (Smo) and, ultimately, to the activation of glioma associated transcription factors Gli1, Gli2, and Gli3, downstream of an intracellular transduction cascade. Gli1 is a powerful oncogene and is upregulated in tumors [214]. Metformin significantly decreased Ptch1, Gli1, and Gli2 protein expression in prostate cancer cell lines in an AMPK-dependent manner [215]. The AMPK activation was suggested to also mediate the metformin-induced downregulation of Shh ligand, Ptch, Smo, and Gli1 expression in breast cancer cells and gastric cancer cells [216,217]. The Hedgehog signaling is also regarded as an early and late mediator of pancreatic cell tumorigenesis. Metformin was found to decrease the Shh ligand expression in multiple pancreatic cancer cell lines [218].

The Shh pathway is also a key pathway in basal cell carcinoma pathogenesis. In agreement with the inhibitory activity of metformin on this pathway as evidenced by preclinical investigations, a recent retrospective study in a population from Iceland showed that metformin was associated to decreased risk of development of this kind of tumor [219].

A recent study in Shh medulloblastoma, a pediatric brain tumor characterized by aberrant activation of the Shh signaling, provided further evidence that biguanides can target the transcriptional activity downstream of the Shh pathway. Moreover, this study unveiled a different molecular mechanism of action, compared with the previously mentioned studies in other tumor types. In detail, in contrast with the supratherapeutic concentrations of metformin tested in various cancer cell types, the authors investigated the effects of micromolar concentrations of phenformin, a biguanide which is more lipophilic and permeable across cell membrane than metformin, and cannot be currently used in humans due to higher risk of lactic acidosis. Micromolar concentration of phenformin, which may be regarded as clinically relevant, exerted an inhibitory effect on tumor growth and Shh signaling that was independent of complex I inhibition and AMPK activation, and linked to the inhibition of mGPDH activity and the increase of intracellular NADH content. The altered redox state leaded to the association between the NADH-dependent transcriptional corepressor CtBP2 and Gli1, thereby inhibiting the Shh signaling [214].

The downregulation of Gli1 expression and transcriptional activity also mediates some effects of metformin in LKB1-wild type non-small cell lung cancer (NSCLC) cell lines. Indeed, the combined treatment of these cells with metformin and MEK inhibitors revealed to be more effective than MEK inhibitors used as single agent at reducing cell proliferation and viability, and reduced the metastatic behavior by affecting the transition from an epithelial to a mesenchymal phenotype, which is mediated by Gli1 over-activation. Moreover, the combined treatment was effective in cell lines showing innate resistance to MEK inhibitors [220]. Actually, the studies in NSCLC cell lines also evidenced that metformin could enhance the MAPK activation through an increased C-RAS/B-RAF heterodimerization, thus suggesting a somewhat unexpected enhancement of the response to growth-stimulatory signals mediated by the RAS/RAF/MAPK pathway when metformin is used as a single agent. These data further support the rationale for testing a co-treatment with metformin and MEK inhibitors or other agents which selectively block molecular targets involved in tumor growth [221,222].

Despite all the studies in cancer cells showed an inhibitory effect of metformin on the Shh signaling, there is some evidence that the action of metformin may be quite different in normal cells and may be affected by extracellular glucose concentrations. In detail, metformin was shown to restore the Hedgehog signaling, which was impaired in endothelial cells exposed to high glucose concentrations, as observed in the retinal vasculature of db/db mice and in cultured HUVEC cells. This effect was suggested to be implicated in the metformin protective effect on the endothelial function. Moreover, these data suggest that the action of metformin on angiogenesis in normal and tumor tissue, needs to be further characterized [223].

Finally, AMPK may also mediate some epigenetic effects of metformin. It has been previously remarked that metformin via AMPK can enhance the activity of SIRT1, a NAD^+^-dependent histone deacetylase, resulting in deacetylation of downstream targets [3,168]. Nevertheless, the AMPK activation by metformin was also shown to induce protein acetylation in various cancer cell types, resulting in altered gene expression [224,225]. To this end, by decreasing the conversion of acetyl-CoA to malonyl-CoA, the AMPK activation may lead to an expansion of the nucleocytosolic acetyl-CoA pool, which is a substrate for histone acetyltransferases, and enhance the acetylation of histone and non-histone proteins [7].

The epigenetic effects of metformin are also relevant to the regulation of the biological machinery of aging. In this context, metformin was shown to directly regulate some epigenetic chromatin modifiers, including demethylase family members [226].

## 8. Metformin Actions on Pituitary Tumor Cells and Gastroenteropancreatic Neuroendocrine Tumor Cells

In the last decade, preclinical research, epidemiological studies, and a few clinical trials have addressed the possible use of metformin as an adjuvant agent in the pharmacological treatment of pituitary adenomas and GEP neuroendocrine tumors, in the case of partial or total resistance to currently approved treatments [17,18,19]. Regarding the role of metformin in the prevention and treatment of endocrine-related cancers, such as prostate, breast, ovary, and endometrial cancer, the reader may refer to a recent review article from Leon–Gonzales et al. [52].

### 8.1. Pituitary Tumors

Pituitary adenomas are usually benign intracranial tumors that are classified as functional (secretory) or non-functional tumors, and categorized based on their mass (micro- or macroadenomas) and the specific pituitary hormone that they release [18,227]. A few years ago, the International Pituitary Pathology Club suggested renaming pituitary adenomas as pituitary neuroendocrine tumors (PitNET) [228]. Actually, no general consensus was obtained and a following international workshop, gathering the representatives of several societies in the expert panel and hosted by the Pituitary Society, drew the conclusion that the term adenoma should be retained, and an improved definition of “aggressive” or “invasive” tumors may represent a significant step forward in the multidisciplinary classification of pituitary adenomas [229].

**Figure 4:**
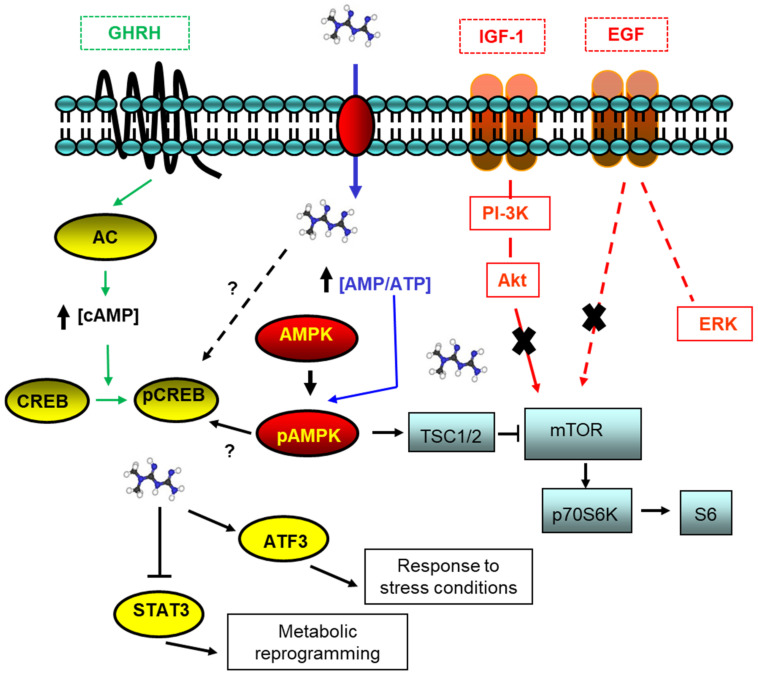
Metformin was shown to reduce cell growth and viability in distinct pituitary adenoma cell lines in vitro. Both AMPK-dependent and -independent actions are involved. Metformin induced AMPK phosphorylation and activation in AtT20 corticotroph cells and GH3 lacto-somatotroph cells. Metformin inhibited the IGF-1R/AKT/mTOR pathway in AtT20 cells, and selectively suppressed the EGF-induced mTOR/p70S6 kinase pathway activation in GH3 cells without affecting ERK1/2 phosphorylation downstream of the same growth factor receptor. The AMPK activation can cooperate to the mTOR pathway inhibition by metformin, but AMPK-independent actions cannot be ruled out. In GH3 cells, metformin was shown to reduce the activity of STAT3, while increasing the activity of ATF3, a transcription factor involved in the response to stress conditions. These actions were not AMPK-dependent. The JAK-STAT3 signaling pathway plays a role in tumor cell metabolic reprogramming, and cooperates with other factors to shift cell metabolism towards aerobic glycolysis. A possible functional interaction between metformin and adenylyl cyclase (AC)-activating stimuli was investigated in GH3 cells. No functional antagonism was seen. On the other hand, metformin tended to further increase CREB phosphorylation. The possible involvement of AMPK was not investigated.

Multiple studies have examined the effects of metformin on the growth and viability of pituitary adenoma cells in vitro [18,19]. Metformin was shown to decrease cell proliferation and cell viability in distinct cell lines (mouse corticotroph AtT20 cells, rat growth hormone/prolactin-secreting GH3 and GH1 cells) [170,171,173]. Regarding the underlying cell signaling pathways, metformin activated AMPK in both AtT20 cells and GH3 cells [171,173]. In AtT20 cells, the drug inhibited the IGF-1R/AKT/mTOR pathway [173]. In GH3 cells, metformin suppressed the EGF-induced mTOR/p70S6 kinase pathway activation. In this regard, it selectively reduced the p70S6 kinase-mediated phosphorylation of S6 ribosomal protein without affecting ERK1/2 phosphorylation, despite both pathways being enhanced by EGF treatment [171]. In GH3 cells, metformin was also shown to inhibit STAT3 and to increase the activity of ATF3, a transcription factor induced by stress conditions. This mechanism was not AMPK-dependent (Figure 4) [170].

Growth hormone-releasing hormone (GHRH) is the main stimulatory factor regulating GH synthesis and secretion from normal somatotroph cells. In these cells, the GHRH receptor (GHRH-R) is coupled to stimulatory heterotrimeric G protein (Gs) and adenylyl cyclase activation. As for human GH-secreting pituitary adenomas, about 40% of patients with sporadic acromegaly harbor guanine nucleotide-binding protein alpha stimulating (GNAS) gene mutations, leading to constitutively elevated cAMP levels [227,230,231].

These premises have suggested investigating a possible functional interaction between metformin and stimuli activating adenylyl cyclase-dependent pathways in pituitary tumor cells, using rat cell lines. Actually, rat GH3 cells do not express the GHRH receptor. Hence, the interaction between metformin and different extracellular stimuli activating adenylyl cyclase, was studied both in native GH3 cells and transfected GH3 cell clones overexpressing the human GHRH-R [171].

Metformin did not affect the adenylyl cyclase activity, albeit it tended to enhance the stimulatory effects of forskolin and GHRH on the cAMP-responsive element binding protein (CREB) phosphorylation. Moreover, metformin enhanced the AMPK activity in native GH3 cells and GH3 cells overexpressing the GHRH receptor treated with forskolin or GHRH, respectively, and inhibited their growth in vitro. In summary, no functional antagonism between metformin and adenylyl cyclase activating stimuli was seen in rat pituitary tumor cells (Figure 4) [171]. On the other hand, this kind of interaction occurs in hepatocytes and is believed to cooperate to the metformin effects on gluconeogenesis. Indeed, it has been previously mentioned that the mild elevation in AMP levels in hepatocytes caused by metformin concentrations of therapeutic relevance, results in the inhibition of adenylyl cyclase activity and, as a consequence, glucagon signaling [78].

The studies examining the metformin actions on human pituitary adenoma cells in vitro are more controversial and still limited. An and coworkers showed that metformin significantly suppressed the cell growth and hormone secretion in primary human GH-secreting adenoma cells [170]. On the other hand, Vazquez–Borrego and coworkers reported on the effects of three biguanides, including metformin, on different types of pituitary tumors. Metformin treatment reduced the cell viability in human ACTH-secreting adenomas and non-functioning adenomas, not so in GH-secreting and prolactin-secreting tumors, and did not affect hormone secretion, despite the high concentration used [35].

An in-lab study and a few case reports have suggested that a combined treatment with metformin and bromocriptine may be effective at controlling hormone secretion and tumor growth in patients with prolactinomas resistant to bromocriptine [232]. Actually, in a more recent pilot study, the metformin addition to ongoing high dose cabergoline treatment in ten patients with cabergoline-resistant prolactinomas, failed to show a consistent effect on serum prolactin levels [22].

The impact of metformin on the lactotroph secretory function in vivo, was also investigated in premenopausal women with prediabetes and hyperprolactinemia and was found to correlate with the vitamin D status. More in detail, the reduction in prolactin levels after metformin administration, correlated with 25-hydroxyvitamin D circulating levels. Actually, it also correlated with the improvement in insulin sensitivity. Since the vitamin D status was not neutral to the glucose and insulin response to metformin [233], it is not possible to say whether the vitamin D-dependent action of metformin on the lactotroph function was direct or indirect, mediated by the improvement in insulin sensitivity.

Acromegaly is a rare disease due to chronic growth hormone (GH) and insulin-like growth factor-1 (IGF-1) excess [234]. Hyperinsulinemia, impaired glucose tolerance, or type 2 diabetes are frequent complications of this syndrome. Metformin is a first-choice treatment for type 2 diabetes and may be administered to acromegalic patients to reduce glycemia and improve insulin sensitivity [235,236]. Therefore, the potential antitumor activity of metformin may offer a rationale for retrospectively and prospectively evaluating the tumor behavior in acromegalic patients with diabetes treated with metformin. Actually, up to now, there is no evidence supporting an effect of metformin on primary tumor progression and biochemical control of the disease in acromegalic patients. Recently, a retrospective, observational, multicenter study was performed to assess whether a metformin treatment might affect the prevalence of secondary neoplasms in acromegalic patients. No significant statistical difference was found between groups, when comparing metformin-treated to -untreated subjects for the presence of a second tumor [21]. On the other hand, a moderate preventive role of metformin on the onset of colon polyps in acromegaly, has been suggested by a distinct exploratory study [237], waiting for confirmation in a larger population.

In summary, despite the tumorigenic potential of hyperinsulinemia, hyperglycemia, and high plasma IGF-1 levels having been generally accepted and metformin contributing to reverse these conditions in acromegalic patients [238,239], there is no clear evidence that a treatment with metformin can reduce the primary tumor growth as well as the onset of secondary neoplasms in acromegalic patients.

Focusing on the in vitro studies, the remarkable effect of metformin on rat pituitary tumor cell proliferation and viability, in contrast with the lack of any response in most human adenoma primary cultures, may suggest that the proliferation rate and the consequent metabolic requirements could play a main role in determining differences in the response to the drug between distinct tumor cell populations. Indeed, primary cultures from human pituitary adenomas may be often characterized by lower proliferation rate compared to rat pituitary cell lines.

In this context, most pituitary tumors are benign pituitary adenomas. Actually, some pituitary adenomas may show an aggressive behavior, with rapid growth, resistance to current therapies and early recurrence. Pituitary carcinomas are rare, malignant tumors with metastatic spread [240]. A recent research article by Onizuka and coworkers remarked the role of metabolic reprogramming in sustaining the differential growth of aggressive pituitary tumors compared with benign adenomas. In fact, the authors showed data which suggest that metabolic reprogramming has an impact on the epigenetic regulation of tumor-promoting genes. In detail, aggressive tumors were characterized by higher levels of glucose transporter GLUT1, increased uptake of glucose and aerobic glycolysis compared with normal pituitary cells and benign adenoma cells. These metabolic features were associated with enhanced histone acetylation and higher proliferation rate. Further studies using the AtT20 cell line, suggested that the glucose-dependent histone acetylation promoted the upregulation of telomerase reverse transcriptase (TERT) expression and, indirectly, the cell cycle-related gene cyclin D1 expression. In summary, the authors concluded that the metabolic reprogramming of glucose metabolism in pituitary tumor cells, may sustain the growth of aggressive tumors through the epigenetic regulation of genes which play a key-role in tumor progression [240].

Finally, this study suggests that both metabolic reprogramming and epigenetic changes may be exploitable targets for the development of novel drug therapies of pituitary tumors, and the sensitivity of pituitary tumor cells to drugs which target cellular bioenergetics, may differ significantly between tumors, based on their proliferation rate and aggressiveness.

### 8.2. Neuroendocrine Tumors

Neuroendocrine tumors (NETs) are classified into different types based on their site of origin. They include gastrointestinal NETs (GI-NETs), pancreatic neuroendocrine tumors (pNETs) and lung carcinoids [13,241]. Diabetes is associated with an increased risk for the development of pNETs and GI-NETs [18,242]. The first clinical evidence of a therapeutic effect of metformin on NETs, have been provided by retrospective studies in diabetic patients suffering from concurrent pNETs. The analysis of the progression free survival (PFS) in diabetic patients treated with metformin compared to diabetic patients not treated with metformin and non-diabetic patients, suggested a direct or indirect antitumor activity of metformin. Moreover, metformin administration improved the efficacy of the pharmacological antitumor therapies based on somatostatin analogs or everolimus [17,187,243,244,245].

A direct inhibitory effect of metformin on NET cell proliferation, viability, and migration capacity has been supported by in vitro studies with various neuroendocrine tumor cell lines representative of distinct NET types. Metformin inhibited the cellular proliferation of neuroendocrine tumor cells of different origins and reduced cell viability in two pNET cell lines (BON-1 and QPG-1). Actually, the effective metformin concentrations in cell growth media exceeded the plasma metformin levels reached in patients treated with safe dosages, just as in studies with pituitary tumor cell cultures [13,187,246,247,248,249].

The cell signaling pathways underlying the growth inhibitory activity of metformin in vitro, may vary significantly between distinct NET cell lines. In this regard, the metformin action in BON-1 cells was associated with a decrease in AKT- and ERK phosphorylation, but not so in QPG-1 cells [247]. Actually, as for ERK phosphorylation, it is worth remarking that the response to metformin may be highly affected by the incubation time in vitro, so that opposite results might be achieved even using the same cell line. In addition, the effect of metformin on AMPK activity in NET cells was shown to vary significantly between distinct cell lines. On the other hand, metformin inhibited the mTOR signaling pathway in all cell lines tested, and promoted glycogen synthase kinase 3 (GSK-3) phosphorylation and inactivation in BON-1 cells (pancreatic tumor), GOT-1 cells (midgut carcinoids), and NCI-H727 cells (bronchopulmonary tumor) [13,246].

GSK-3 is a serine-threonine kinase involved in the control of cell metabolism and multiple cellular functions, such as cell proliferation, autophagy, programmed cell death, and may represent a therapeutic target in NETs. In NET cell lines, the GSK-3 inhibition was associated with inhibition of EGF receptor (EGFR) signaling pathway and mTOR inhibition [13,250].

Finally, in QPG-1 cells, metformin was shown to upregulate the aryl hydrocarbon receptor-interacting protein (AIP). The outcome of AIP silencing in these cells, suggest the involvement of this factor in the antitumorigenic effect of metformin, which is again related to the mTOR pathway downregulation [248,249].

Cell signaling studies in cultured cells and the analysis of clinical data from patients enrolled in retrospective studies have both contributed to suggest some intracellular targets of metformin, which may be responsible for its anticancer activity against pNETs (Table 4).

The insulin receptor/IGF-1 receptor/PI3K/AKT/mTOR pathway is believed to play a central role in pNET cell growth and proliferation [187,249,251,252]. Indeed, the mTOR inhibitor everolimus and the multi-tyrosine kinase inhibitor sunitinib demonstrated anticancer activity and prolonged the median PFS in pNET patients pre-treated with somatostatin analogs [187,253,254]. The ability of metformin to inhibit this pathway in an AMPK-dependent and -independent manner is likely to cooperate to its antitumor effects and may account for a synergistic activity between mTORC1 inhibitors and metformin. More precisely, the mTOR-p70S6 kinase signaling pathway plays a main role in the cell growth regulation downstream of growth factor receptors. A drawback in suppressing the mTOR signaling in tumor cells, is the removal of the negative feedback loop mediated by p70S6 kinase, thus leading to the reactivation of PKB/Akt and ERK1/2 pathways downstream of growth factor receptors (i.e., insulin and IGF-1 receptors). This event can limit the efficacy of direct mTORC1 inhibitors, such as everolimus, with the possible occurrence of tumor cell escape from therapy [255,256,257,258]. Metformin is able to affect the mTOR signaling by different pathways, and evidence has been provided that a combined treatment with metformin and everolimus may reduce the risk of tumor cell escape from therapy. Indeed, Vitali and coworkers showed that the combined treatment was more effective than monotherapy in inhibiting pNET cells in vitro. Moreover, the authors also developed a model of everolimus-resistant cells and proved that metformin maintained its effects in these cells [248].

The effects of metformin on the mTOR signaling pathway activation in pNET cells, may be also indirect and mediated by the systemic action on glucose metabolism and plasma insulin levels. Actually, in the previously mentioned retrospective studies [17,243,244], the glycemic status of patients was not independently associated with the response to antitumor therapies, as assessed by the analysis of PFS. These data argue against the hypothesis that the effect of metformin on pNET progression, may be primarily mediated by the ability to reduce glycemia and insulinemia [244]. However, other systemic effects may be implicated in metformin actions on pNET progression.

In patients with advanced pNETs treated with everolimus, the rapid onset of hypertriglyceridemia within few months of treatment and high cholesterol levels, were found to be associated with lower PFS. Moreover, high intra-tumor levels of acetyl-CoA carboxylase A1 (ACC1), were found to correlate with lower efficacy of everolimus [245]. Metformin does not significantly affect the glucose and lipid metabolism in patients with normal baseline profiles but, according to some studies, it has an impact on lipid metabolism in type 2 diabetic patients with a metabolic syndrome profile [187,259]. Moreover, metformin is generally accepted to inhibit the ACC1 activity within cells, in AMPK-dependent manner [197]. Based on these premises, Vernieri and coworkers drew the conclusion that the observed association between metformin administration and longer progression-free survival in patients with pNETs, as well as the synergistic interaction between metformin and everolimus, can also be explained by direct and indirect effects of metformin on tumor cell lipid biosynthesis and systemic lipid metabolism, respectively [17,187,245].

## 9. Conclusions and Perspectives

The in vitro studies have suggested that metformin is equally effective in reducing cell proliferation and/or viability of pituitary adenoma cells and various types of neuroendocrine tumor cells. The effects were seen when metformin was used at concentrations in culture media which exceed the therapeutic concentrations for its currently approved clinical applications.

As for clinical evidence, a few pilot studies and retrospective studies did not confirm the results of the in vitro studies. Indeed, the efficacy of metformin was found to be restrained to pNETs. In these tumors, metformin can affect the tumor progression and improve the response to current drug therapies. The strongest evidence exists for combining metformin with everolimus, which could synergize at cellular and systemic levels. Actually, prospective studies on the antitumor activity in patients with pituitary or neuroendocrine neoplasia are not available yet.

The discrepancy between in vitro data and clinical observations, may be linked to the lower metformin concentrations in the plasma of patients during treatment (approximately, 10^−5^ M) compared to the effective concentrations in vitro (10^−4^–10^−3^ M). Nevertheless, if the effects of metformin on pancreatic neuroendocrine tumors were confirmed in prospective studies, it would be important to find out which factors make pancreatic neuroendocrine tumors more responsive to metformin than other endocrine neoplasms.

Current knowledge suggests considering the following factors.

At a cellular level, the contribution of the PI3K/AKT/mTORC1/p70S6K signaling pathway in sustaining the growth of a specific tumor in vivo may contribute to determine its response to metformin therapy. To this end, a deregulation of this pathway is commonly involved in pNET tumorigenesis [243].

The ability for tumor cells to efficiently engage adaptive programs and escape the energetic stress conditions caused by metformin, can affect the therapy efficacy. The cell adaptive responses include increased glycolysis, reductive carboxylation, glutamine metabolism in the short-term, and changes in enzyme expression levels in the long-term. In this context, the AMPK activity may play two opposite roles. AMPK signaling contributes significantly to the adaptive responses of cells to metformin treatment. Hence, an impaired AMPK activity may limit the chance of tumor cell escape from therapy. On the other hand, activated AMPK also mediates the growth-restraining effects of metformin in some tumor types, including the action on tumor cell lipid metabolism.

According to some in-lab studies, also the expression levels and the post-translational regulation of the PDH complex activity, may be central in the tumor cell response to metformin. Actually, more in-depth studies on this topic are required.

A quantitative analysis of intracellular metabolic intermediates after incubation with a given metabolic substrate, by the use of liquid chromatography-mass spectrometry-based stable isotope tracer, might help to elucidate the fate of pyruvate in distinct tumor cell populations in vitro after treatment with metformin, at a metabolic branch point linking various anaplerotic and catabolic pathways and heavily affecting ATP and reducing equivalents levels.

At a systemic level, the antitumor activity of metformin should be evaluated in the light of the involvement of systemic factors, hormones, and metabolites, in sustaining tumor growth. In this regard, metformin could induce changes in glucose metabolism and, consequently, plasma insulin levels and free IGF-1 levels. More in detail, metformin is expected to reduce glucose availability and insulin/IGF-1-mediated activation of anabolic and mitogenic pathways in tumor cells. As a consequence, it may be important to elucidate whether hyperglycemia and hyperinsulinemia are independent risk factors for the development and growth of a given endocrine tumor. Moreover, it has been already remarked that metformin can also reverse some alterations of systemic lipid metabolism which may affect the tumor responsiveness to therapy.

The efficacy of metformin in improving a parameter such as PFS, may also depend on targeting the interaction between tumor cells and adjacent structures and the metastatic spread of tumors. In summary, the biological traits and clinical behavior of a given endocrine tumor can be factors affecting its response to metformin in vivo, which cannot be easily replicated in vitro using immortalized cell lines or even primary cultures.

Finally, some limitations inherent to the in vitro studies should be further discussed.

Metformin has been revealed to be effective in vitro at concentrations ranging from 10(−4) to 10(−3) M. Based on the plasma levels of metformin when used to treat type 2 diabetes, these concentrations exceed the therapeutic concentrations and may be toxic in vivo. However, metformin can accumulate within cells and in mitochondria, thus reaching intracellular concentrations which exceed the plasma concentrations at the steady state [13,59,60]. Moreover, although used in vitro at supratherapeutic concentrations, metformin is still a valuable experimental tool to characterize the metabolic profile of a given tumor cell population, and hence its vulnerability to other specific agents targeting cell metabolism as well as its dependence on specific metabolic substrates for growth.

Due to its direct non-site-specific effects on catabolic and anabolic pathways within cells, metformin can simultaneously act on different cell populations, which may include distinct subpopulations of tumor cells showing differential sensitivity to the drug, and normal cells in the tumor microenvironment and surrounding structures (stromal cells, endothelial cells, fibroblasts). Hence, metformin is also expected to alter the release of metabolites and signals from both normal and tumor cells, thus affecting the crosstalk between the distinct cell populations in the tumor microenvironment [260,261]. In summary, the heterogeneity of cells within tumor mass and cell-context variability may account for differential responses to metformin between distinct tumors or distinct tumor types. In this regard, monocultures of a given tumor cell population are still a useful but not fully representative tool to investigate the actual efficacy of metformin, and further studies using co-cultures or three-dimensional cultures may be a necessary step forward.

## Figures and Tables

**Table 1:** Summary of the debated intracellular targets and actions of metformin underlying the inhibition of gluconeogenesis in hepatocytes.

Intracellular Actions	Mechanism Mediating Gluconeogenesis Inhibition	Reports Questioning the Suggested Mechanism
AMPK activation	Transcriptional regulation Downregulation of *G6pc* and *Pck1* gene expression	34,73,81,82
Inhibition of mGPDH	Inhibition of glycerophosphate shuttle Redox-dependent inhibition of gluconeogenesis	39,69,86,88
Changes in the intracellular levels of metabolites(AMP, fructose 1,6-P_2_, fructose 2,6-P_2_)	Allosteric or substrate-dependent regulation of gluconeogenic or glycolytic enzymes Redox-independent inhibition of gluconeogenesis	
Changes in the intracellular levels of AMP	Inhibition of adenylate cyclase Inhibition of glucagon signaling	73,75,81,82

**Table 2:** Summary of metformin actions in tissues other than liver and related to possible beneficial effects on glucose homeostasis and energy balance.

Target	Intracellular Actions	Effects on Metabolic Health
Skeletal muscle	Increased basal glucose uptake	Possible impact on glucose utilization and plasma glucose homeostasis
	Altered BCAA catabolism	
Intestine	Increased glucose uptake	Impact on glucose homeostasis and food intake
	Increased lactate production, possibly associated with a futile enterocyte-hepatocyte futile cycle (lactate-glucose)	Some side-effects associated with metformin treatment in humans
	Reduced bile acid absorption, with consequences on GLP-1 and peptide YY secretion	
Gut microbiota	Changes in the relative abundance of bacterial strains, possibly associated with an impact on SCFA production	Impact on glucose homeostasis, appetite and body weight gain
Intestine and kidney	Increased expression and release of GDF15 (increased circulating levels)	Impact on energy balance and body weight gain
Medio-basal hypothalamus	Decreased AMPK activity	Impact on food intake

**Table 3:** Cell bioenergetics related to congenital or acquired PDH complex deficiency.

Congenital PDH Complex Deficiency	Aging	Cancer
Aerobic glycolysis Increased lactate production Decreased flux though TCA cycle Increased oxaloacetate synthesis through PC activity Overexpression and stabilization of HIF1α, leading to further downregulation of PDH activity and oxidative phosphorylation, and increased glycolysis [129,131,132,133,134]	Increased long chain fatty acid oxidation in skeletal muscle Increased acetyl-CoA and NADH levels, leading to increased PDK activity Decreased insulin-mediated tonic stimulation of PDPs The outcome is a decrease of the PDH complex activity Increased lactate production [129,137,138]	Upregulation and stabilization of HIF1α Increased glucose uptake, glycolysis, and lactate production Increased extrusion of lactate and protons Increased PDK activity, leading to inhibition of the PDH complex activity Decreased oxidative phosphorylation Increased use of glutamine as energy substrate, upregulation of SIRT4 lipoamidase activity, leading to further downregulation of PDH complex activity [83,129,137,138,139,140,141]

**Table 4:** Cell signaling studies in cultured cells and the analysis of clinical data from patients enrolled in retrospective studies have both contributed to suggest some intracellular targets of metformin and systemic actions, which may be responsible for its anticancer activity against pNETs.

Molecular Targets within Cells	Systemic Actions
Insulin receptor or IGF-1R/PI3K/AKT/mTOR signaling pathway (inhibition) GSK-3 (phosphorylation and inhibition) AMPK (phosphorylation and activation) AIP protein (upregulation) ACC1 (AMPK-mediated inhibition)	Increased insulin sensitivity Decreased glycemia and insulinemia Impact on lipid metabolism (in type 2 diabetic patients with a metabolic syndrome profile only)

## Data Availability

Not applicable.

## Abbreviations

AC	adenylyl cyclase
ACC1	acetyl-CoA carboxylase A1
AIP	aryl hydrocarbon receptor-interacting protein
AMPK	AMP-activated protein kinase
AMP	adenosine monophosphate
ATM	ataxia-telangiectasia mutated kinase
ATP	adenosine triphosphate
CNS	central nervous system
CREB	cyclic AMP response element binding protein
CRTC2	CREB regulated transcription factor 2
ERK	extracellular signal-regulated kinases
ETC	electron transport chain
ERRα	estrogen receptor relatedα
FBP1	fructose bisphosphatase-1
FOXO1	forkhead box O1
FOXO3	forkhead box O3
fructose 1,6-P_2_	fructose 1,6 bisphosphate
fructose 2,6-P_2_	fructose 2,6 bisphosphate
GLP-1	glucagon-like peptide-1
GDF15	growth differentiation factor-15
GHRH	growth hormone-releasing hormone
GSK-3	glycogen synthase kinase 3
HIF1α	hypoxia inducible factor 1 subunit α
HNF4α	hepatocyte nuclear factor 4α
LDH	lactate dehydrogenase
MAPK	mitogen-activated protein kinase
MATE	multidrug and toxin extrusion proteins
MCT1 MCT4	H^+^⁄ lactate monocarboxylate transporterH+⁄ lactate monocarboxylate transporter
MEK	mitogen-activated protein kinase kinase
mGPDH	mitochondrial glycerophosphate dehydrogenase
mTORC1	mammalian target of rapamycin complex 1
NADH	nicotinamide adenine dinucleotide
NET	neuroendocrine tumor
NRF-1	respiratory factor 1
NRF-2	nuclear factor erythroid 2-related factor
OCT1	organic cation transporter subtype 1
PC	pyruvate carboxylase
PCOS	polycystic ovary syndrome
PDH	pyruvate dehydrogenase
PDHE1α	pyruvate dehydrogenase E1αprotein
PDK	pyruvate dehydrogenase kinase
PDP	pyruvate dehydrogenase phosphatase
PEP	2-phosphoenolpyruvate
PEPCK	phosphoenolpyruvate carboxykinase
PFK1	phosphofructokinase-1
PFS	progression free survival
PI3K	phosphoinositide 3-kinase
PGC1-α	peroxisome proliferator-activated receptor gamma coactivator 1-alpha
PMAT	membrane monoamine transporter
PPARα/δ	peroxisome proliferator-activated receptor α/δ
p70S6K	p70 S6 protein kinase
ROS	reactive oxygen species
Shh	Sonic Hedgehog
STAT3	signal transducer and activator of transcription 3
SIRT	sirtuin
SREBP1	sterol regulatory element-binding protein 1
TCA cycle	tricarboxylic acid cycle
TSC	tuberous sclerosis complex
